# Design and Evaluation of Quinoline-derived Fluorophores for Labeling Amyloid Beta 1–42 in Alzheimer’s Disease

**DOI:** 10.1021/acsomega.5c07481

**Published:** 2026-01-29

**Authors:** Alma Victoria Sánchez-Mendoza, Rosa Angeles Vázquez-García, Víctor Castaño, Mónica A. Torres-Ramos, Alan Hipólito Juárez-Solano, Raúl Horacio Camarillo-López, Martha Cecilia Rosales-Hernández

**Affiliations:** a Laboratorio de Biofísica y Biocatálisis, Sección de Estudios de Posgrado e Investigación, Escuela Superior de Medicina, 27740 Instituto Politécnico Nacional, Plan de San Luis y Díaz Mirón s/n, Ciudad de México 11340, México; b División de Ingeniería en Nanotecnología, Universidad Politécnica del Valle de México, Av. Mexiquense, esq, Av. Universidad Politécnica s/n, Villa Esmeralda, Tultitlan, Estado de México 54910, México; c Área Académica de Ciencias de la Tierra y Materiales, Universidad Autónoma del Estado de Hidalgo, Cd. Universitaria, Pachuca de Soto, Hidalgo 42184, México; d Centro de Física Aplicada y Tecnología Avanzada, Universidad Nacional Autónoma de México, Boulevard 3001, Santiago de Querétaro, Querétaro 76230, México; e Dirección de Investigación, Instituto Nacional de Neurología y Neurocirugía Manuel Velasco Suárez, Insurgentes sur 3877, La Fama, Tlalpan, Ciudad de México 14269, México; f Centro de Investigación sobre el Envejecimiento, Centro de Investigación de Estudios Avanzados, Calz. de los Tenorios 235, Coapa, Granjas Coapa, Tlalpan, Ciudad de México 14330, México

## Abstract

Amyloid beta (Aβ) is a key biomarker in Alzheimer’s disease, driving the formation of senile plaques that contribute to neuronal death within a complex etiology. Typically, most treatments begin at advanced stages, when irreversible brain atrophy has already occurred; therefore, early diagnosis is essential for effective intervention. Several probes based on the conventional donor−π–acceptor (D−π–A) structural motif have been developed as diagnostic tools, yet few have reached clinical trials. Alternatively, quinoline-based fluorescent compounds with push–pull structures and aggregation-induced emission properties show enhanced fluorescence in the aggregated state due to restricted intramolecular motion (RIM). Accordingly, four quinoline derivatives—2QnCN, 3QnCN, 3QnB, and 4QnBB—were synthesized using standard methods, including benzoxazole segments and a cyano (−CN) group. They were chemically and optically characterized, and their photophysical properties were calculated. Theoretical analyses include band gap estimation and visualization of intramolecular charge transfer. Molecular docking was also performed to assess binding with the Aβ_1–42_ pentamer (PDB: 2BEG), identifying 3QnCN as the most promising candidate with a binding energy of–11.9 kcal/mol. Cytotoxicity was tested using the MTT assay to determine the optimal working concentration. The fluorescence intensity of 3QnCN in PC12 cells was quantified, and confocal microscopy confirmed its effectiveness in labeling Aβ_1–42_.

## Graphical abstract

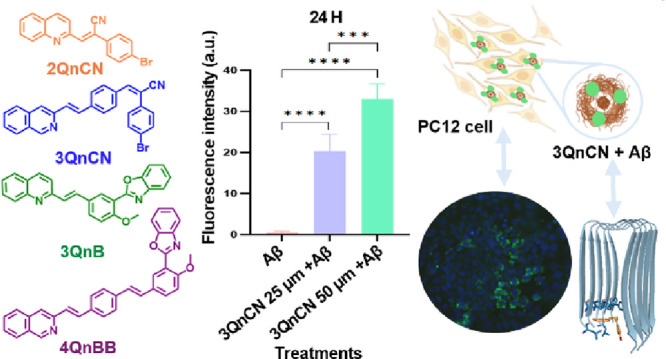

## Introduction

Alzheimer’s disease (AD) is a progressive neurodegenerative disorder that impairs brain regions responsible for cognitive functions, including memory, language, and problem-solving. The principal pathological features driving disease progression include Aβ deposition, formation of neurofibrillary tangles (NFTs), activation of neuroinflammatory pathways, and cholinergic deficits.^1^ AD is increasingly recognized as a multifactorial disorder, with Aβ accumulation representing only one element of a broader pathological process. Additional factors, such as tau protein aggregation, neuroinflammation, oxidative stress, and mitochondrial dysfunction, are strongly associated with the cognitive decline observed in the later stages of the disease.^2–4^ Nevertheless, Aβ is still regarded as a potential early trigger in disease progression.^5,6^

Recent advances in AD research emphasize prevention of Aβ oligomerization, neutralization of toxic oligomer through immunotherapy, and inhibition of enzymes that generate these species.^7,8^ Detection and visualization of amyloid plaques, key biomarkers of AD, are essential for diagnosis and monitoring disease progression. Fluorescence imaging technology holds much promise for *in vivo* applications, but only a limited number of fluorescent probes, such as polymer dots and small organic molecules, have advanced to clinical trials.^9–11^ This limitation arises partly from techniques such as positron emission tomography (PET), which remain costly and technically demanding.^12–14^ The electron-donating and electron-accepting properties of quinoline make it an attractive scaffold for designing new fluorescent probes, offering broad conjugation possibilities and enhanced detection sensitivity.^15,16^

In this context, the *in vivo* detection of Aβ plaques is particularly relevant as an early indicator, given evidence that their formation precedes the onset of clinical symptoms in patients suspected of having AD.^17,18^ Aβ_1–42_ is now widely recognized as a key biomarker of AD,^19,20^ and imaging its aggregates has become indispensable for diagnosis and disease monitoring and for therapeutic evaluation.^21–23^ Several design approaches have been proposed, with particular emphasis on small molecules that bind to the hydrophobic regions of Aβ_1–42_.^24^ During preclinical or clinical assessment, AD diagnosis typically involves extensive behavioral testing combined with neuroimaging techniques such as PET; however, this procedure is costly and time-consuming.^13,14^ At present, a definitive diagnosis of AD is possible only through post-mortem histopathological analysis of the brain tissue.^25–27^

Accordingly, *in vivo* imaging of Aβ is especially valuable for detecting individuals at risk and identifying early stage AD, enabling timely and effective intervention before the onset of irreversible neuronal atrophy. Optical imaging technology provides several advantages as a noninvasive and safe procedure, offering high spatial resolution, strong visualization capacity, rapid performance, and cost-effectiveness.^28^ Consequently, they have been widely applied in biomolecule detection, metabolic tracking of drug distribution, and disease diagnosis.^7,29,30^

In the context of AD diagnosis, Congo red (CR), thioflavin T (ThT), and related dyes have long been employed for post-mortem histological staining of amyloid fibrils.^31^ Recent advancements have enabled the development of small molecules that interact with Aβ plaques at site B, a region encompassing the hydrophilic N-terminal and the central hydrophobic domain of the Aβ sequence^32^ These interactions are associated with phenomena such as wavelength shifts, altered spectrum shifts, and enhanced fluorescence emission upon binding.^33^ Molecules designed with a donor−π–bridge–acceptor (D-π-A) architecture show a pronounced increase in fluorescence when bound to Aβ aggregates^34^ Likewise, polyene scaffolds extend the π-conjugated system, producing emission at longer wavelengths.^35^ To achieve effective structural design, various functional groups have been explored: cyano and dicyanomethylene moiety as electron acceptors,^34,36^ benzene rings as electron donors linked by π bonds,^37^ and quinoline, typically serving as an acceptor unit. Xie et al. (2021)^38^ reported fluorophores based on a donor–acceptor framework, constructed by linking dibutyl-2-naphthylamine as a donor with quinoline as an acceptor via a double bond. These fluorophores exhibited strong emission properties. As a result, a selective increase in binding affinity and fluorescence toward Aβ oligomers was observed when examining interactions with different Aβ species, including monomers, oligomers, and fibrils. These findings indicate promising potential for AD therapy through inhibition of Aβ self-aggregation, neuroprotective effects against Aβ-induced toxicities, and suppression of reactive oxygen species generation.

In the past decade, there has been increasing interest in and progress on AIE molecules first reported in 2001.^39^ These molecules represent a novel class of materials with important applications in environmental and health monitoring, disease diagnosis, bioactive molecule detection, and electronic devices.^40^ Unlike conventional fluorophores that undergo aggregation-caused quenching (ACQ), AIE molecules provide clear advantages for studying protein aggregates linked to diseases. These display intense fluorescence in the aggregated state, in contrast to their weak or lack of fluorescence when isolated, thereby overcoming limitations such as severe self-quenching, poor photostability, and small Stokes shift typical of conventional ACQ fluorophores.^41^ Substantial advances have been reported in various AIE molecules with different mechanisms, including tetraphenylethene (TPE), tetraphenylbutadiene (TPBD), quinoline–malononitrile (QM), and hexaphenylsilole (HPS), designed via RIM.^41,42^ Despite the potential, research in the field remains at an early stage with limited exploration.

Accordingly, the synthesis of four quinoline compounds containing benzoxazole segments and a CN group was investigated. Their photophysical properties were assessed along with theoretical calculations and molecular docking studies. Among the synthesized compounds, 3QnCN was notable for its excellent photophysical characteristics, including a higher molar extinction coefficient and superior quantum yield compared to its analogs. Its labeling ability was confirmed by fluorescence microscopy, which revealed a green signal around the nucleus of PC12 cells stimulated with Aβ_1–42_.

## Results and Discussion

### Chemical synthesis of 2QnCN, 3QnCN, 3QnB, and 4QnBB

Compounds 2QnCN, 3QnCN, 3QnB, and 4QnBB were synthesized as shown in Figure 1 using modified versions of the Knoevenagel,^43^ Wittig,^43^ Heck,^43^ and aldol condensation reactions, respectively.^44^ For 3QnB and 4QnBB, the 2Ben segment was prepared following the method described by López-Ruiz et al. (2011).^45^ Compound 2QnCN was isolated as a white powder with a 95% yield (0.10 g), 3QnCN as a yellow powder with a 97% yield (0.049 g), 3QnB as a brown powder with a 62% yield (0.029 g), and 4QnBB as a brown powder with a 47% yield (0.017 g).

**Figure 1:**
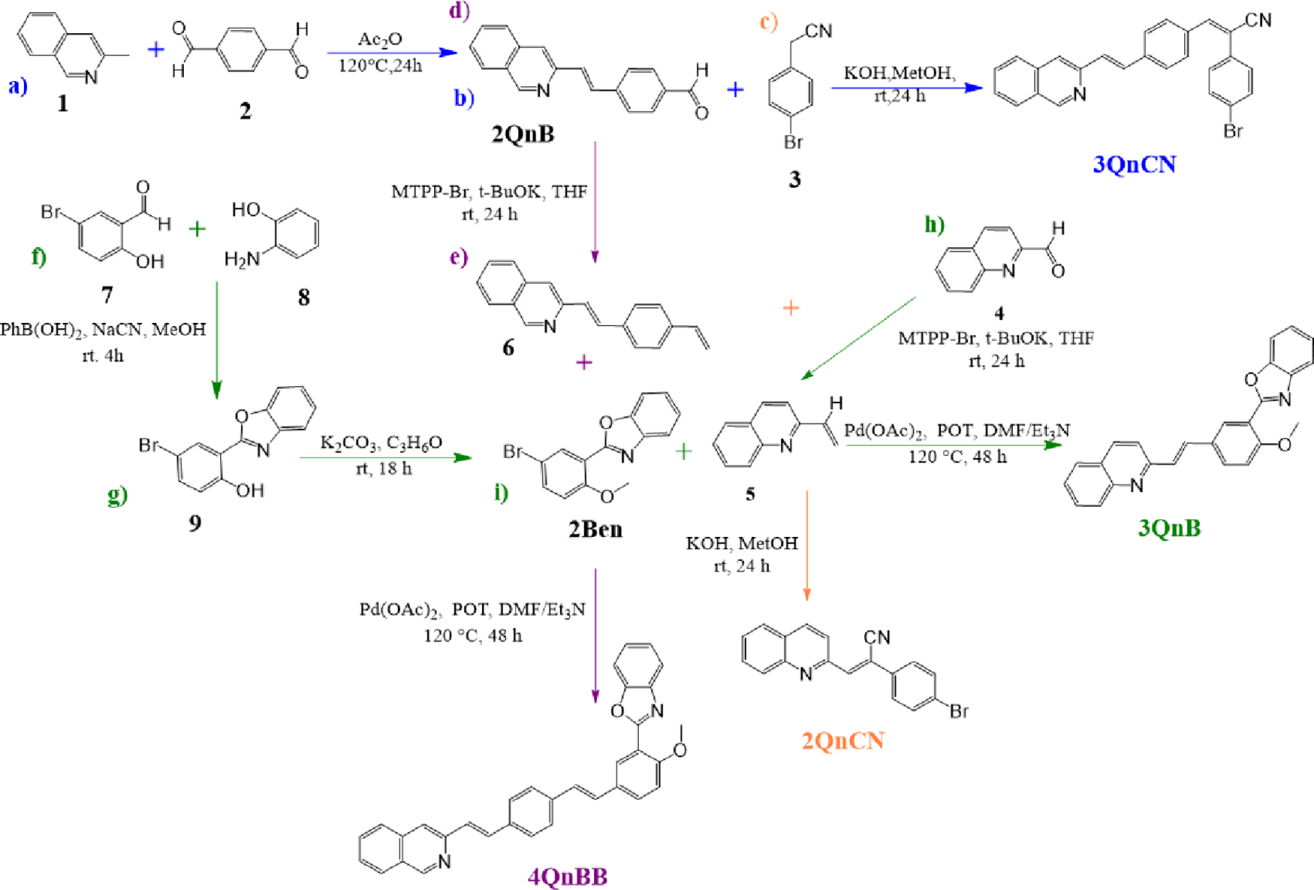
Chemical synthesis pathway of compounds 2QnCN, 3QnCN, 3QnB, and 4QnBB.

### Characterization of 2QnCN, 3QnCN, 3QnB, 4QnBB, and Optical Properties in Solution

The absorption bands of 2QnCN (366 nm), 3QnCN (378 nm), 3QnB (363 nm), and 4QnBB (396 nm), corresponding to π–π* electronic transitions,^46,47^ are shown in Figure 2(red spectrum). The observed shift reflects the degree of conjugation within each extended electronic system; in 3QnCN and 4QnBB, this effect also contributed to a decrease in their optical band gap (Eg_Opt_).

**Figure 2:**
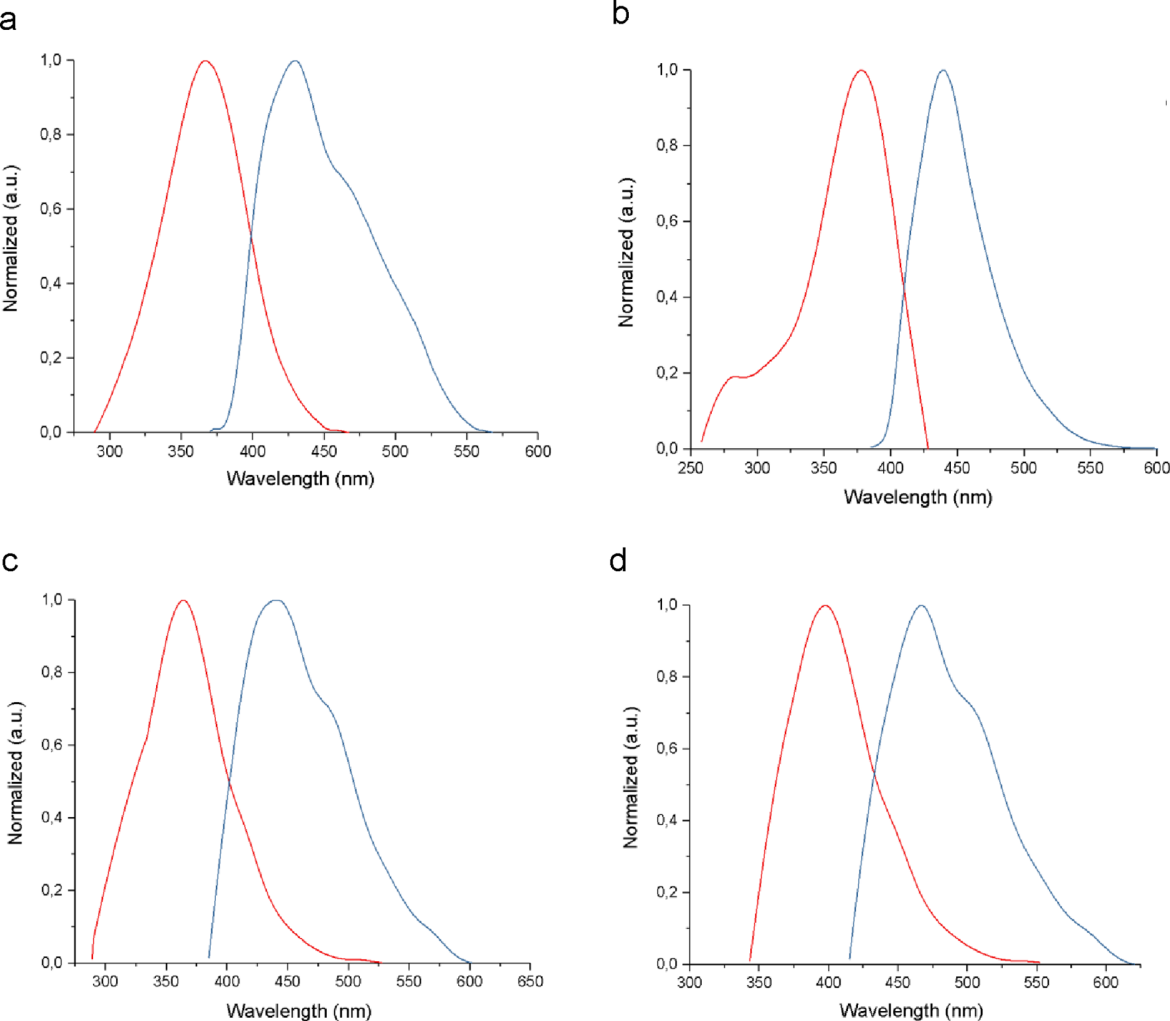
Absorption and fluorescence spectra in dimethyl sulfoxide (DMSO), (a) 2QnCN; (b) 3QnCN; (c) 3QnB; (d) 4QnBB. Red curves represent absorption spectra, and blue curves represent fluorescence spectra.

The fluorescence emission spectrum of 2QnCN spans from violet to green (Figure 2a), whereas that of 3QnCN extends into the yellow region (Figure 2b). For the benzoxazole-containing compounds (3QnB and 4QnBB), the emission spectra range from violet to orange (Figure 2c, 2d). The emission bands are observed at 429 nm for 2QnCN, 439 nm for 3QnCN, 374 nm for 3QnB, and 389 nm for 4QnBB (blue spectrum).

Table 1 summarizes the optical properties of 2QnCN, 3QnCN, 3QnB, and 4QnBB. The highest molar extinction coefficient (ε) was observed for 3QnCN at 2.2 × 10^4^ M^–1^ cm^–1^ with a maximum absorbance at 378 nm, whereas 4QnBB exhibited an ε of 4.5 × 10^4^ M^–1^ cm^–1^ with maximum absorbance at 396 nm. The Stokes shift data indicate that the excited state of 3QnCN undergoes fewer nonradiative energy losses prior to fluorescence emission.^7,47^ The fluorescence quantum yield (φ) of 3QnCN, measured using quinine sulfate as a reference, was determined to be 0.02.

**Table 1:** Optical Properties of 2QnCN, 3QnCN, 3QnB, and 4QnBB in Dimethyl Sulfoxide (DMSO)

molecule	λ_abs max_ (nm)	εx10^4^ (M^–1^.cm^–1^)	***E***_***g o* ´** ***ptico***_(eV)	λ_onset_ (nm)	fwhm (nm)	λ_fluo max_ (nm)	Φ	Stokes shift (cm^–1^)
2QnCN	366	3.2	2.2	425	89	429	0.001	63
3QnCN	378	2.2	2.4	426	60	439	0.02	61
3QnB	364	3.9	2.3	433	103	440	0.01	77
4QnBB	397	4.5	2.6	465	93	469	0.01	70

The emission spectra further reveal distinct profiles: compound 3QnCN (Figure 2b) shows a single peak, whereas 2QnCN, 3QnB, and 4QnBB (Figure 2a,c,d, respectively) each display a main emission peak accompanied by a shoulder at higher wavelengths.

Considering the design of molecules containing quinoline groups—well-known for their strong chromophoric properties^48^—together with the cyano (CN) group, widely applied in marker design,^49,50^ the four quinoline-derived compounds display fluorescence maximum between 350 and 450 nm, consistent with previously reported marker compounds.^41^ The quantum yields are below 0.1, a characteristic feature of this class of molecules, as such values can influence fluorescence intensity changes or redshifts upon binding to Aβ species.^35^ The molar extinction coefficient (ε) reflects the efficiency of a compound in absorbing electromagnetic radiation at a given wavelength. Among the synthesized derivatives, 3QnCN shows the highest ε, indicating superior photon absorption capacity in the studied spectral region. This enhanced performance can be attributed to the cyano group (−CN) in its structure, which acts as a strong electron acceptor, promoting delocalization across the conjugated system. As a result, the probability of π–π* electronic transitions increases, directly enhancing ε.

By contrast, the other derivatives lack substituents with comparable electron-accepting strength and therefore exhibit lower extinction coefficients. Overall, these findings suggest that 3QnCN possesses the most favorable electronic configuration within the series for maximizing light–matter interactions.

From an applied standpoint, a higher molar extinction coefficient means that lower biomarker concentrations are sufficient to generate a detectable optical signal, an advantage in bioimaging and molecular detection under physiological conditions. In this respect, 3QnCN emerges as the most promising candidate among the analogs.

### Theoretical Studies

The molecular design of 2QnCN and 3QnCN incorporated a quinoline segment as an acceptor a central phenylvinylidene donor fragment, and a CN group acting as an additional acceptor, forming a push–pull electron-attractive architecture, and for 3QnB and 4QnBB, a benzoxazole segment containing electron-donating and electron-accepting groups was introduced. Geometry optimization performed with DFT revealed partial planarity of the main π-conjugated system (Figure 3). The calculated electron density maps indicate that the Highest Occupied Molecular Orbital (HOMO) is distributed along the primary backbone of 2QnCN and 3QnCN (Figure 3a,b).

**Figure 3:**
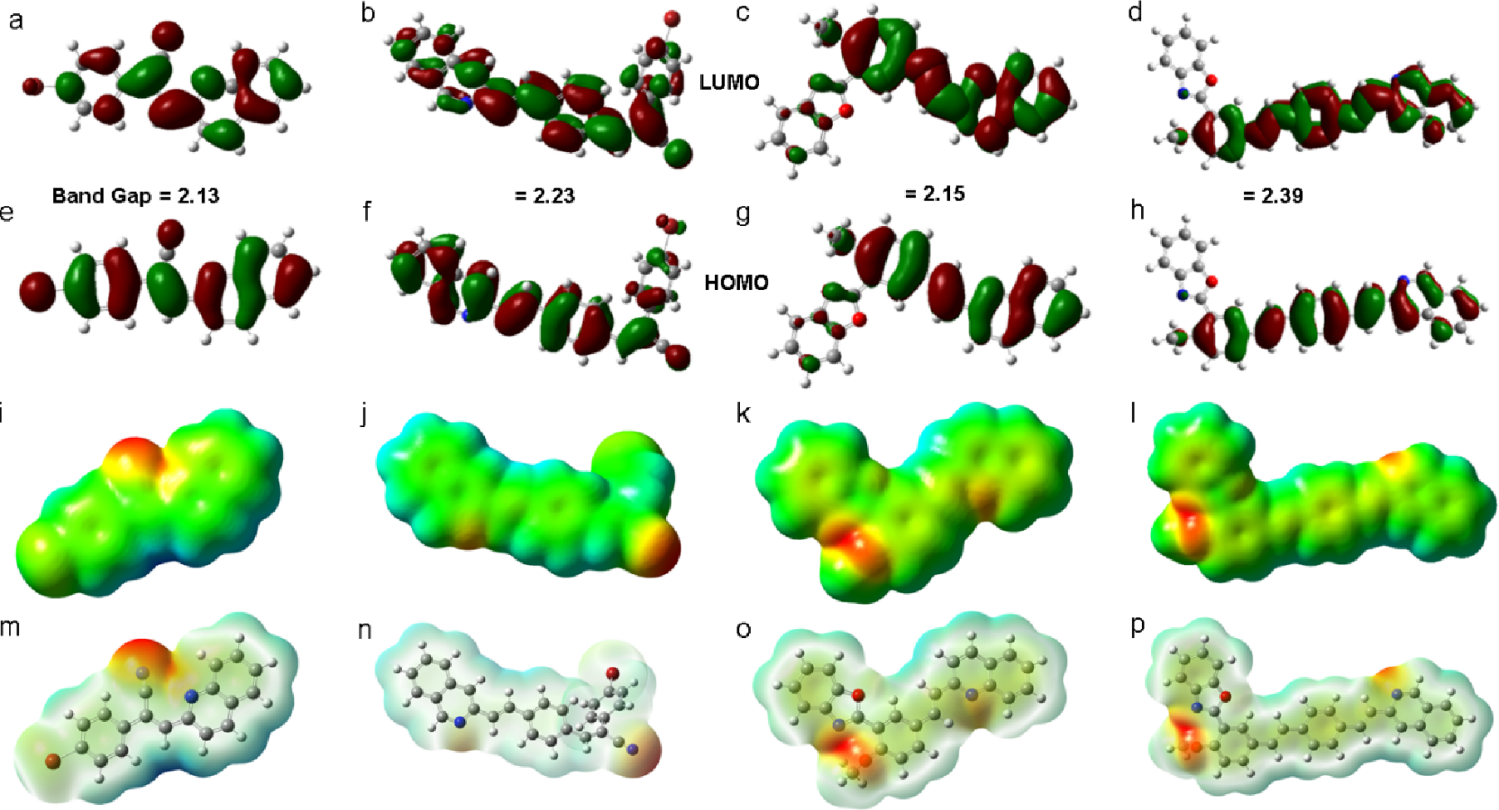
Optimized conformations of (a) 2QnCN, (b) 3QnCN, (c) 3QnB, and (d) 4QnBB calculated by DFT B3LYP/6–31 (d); electron density distributions of (e) 2QnCN, (f) 3QnCN, (g) 3QnB, and (h) 4QnBB.

The Lowest Unoccupied Molecular Orbital (LUMO) electron density is mainly localized on the quinoline rings. However, in 3QnB and 4QnBB (Figure 3c,d), it becomes interrupted by the benzoxazole segment for the HOMO and LUMO, thereby hindering intermolecular charge transfer. These results confirm that quinoline serves as an excellent electron acceptor unit.^43^ Time-dependent DFT (TD-DFT) further supports a possible HOMO–LUMO transition (Table 2).

**Table 2:** Electronic Transitions Obtained from BHandHLYP/6-31G­(d,p) Calculated for 2QnCN, 3QnCN, 3QnB, and 4QnBB

molecule	Λ abs (nm)	E(tr) (eV)	OS (f)	MO/character
2QnCN	367.88	3.29	0.79	H → L (98%)
	361.93	3.42	0.006	H – 2 → L (99%)
	353.16	3.51	0.11	H – 1 → L (91%), H → L + 1 (6%)
3QnCN	380.41	3.00	1.32	H → L (99%),
	350.58	3.54	0.02	H – 1 → L + 1 (47%)
	333.10	3.72	0.10	H – 1 → L (42%), H – 1 → L + 1 (36%), H → L + 2 (13%), H – 2 → L (6%)
3QnB	362.00	3.46	0.92	H → L (89%)
	336.97	3.68	0.07	H-1 → L (26%), H → L + 1 (62%), H → L (6%)
	319.78	3.88	0.03	H – 2 → L (63%), H – 1 → L (10%), H → L + 2 (20%)
4QnBB	395.78	3.00	2.01	H → L (99%)
	363.66	3.41	0.05	H → L + 1 (86%), H – 1 → L (8%)
	358.94	3.45	0.15	H → L + 2 (89%), H – 2 → L (4%), H – 1 → L (2%)

Summary of electronic transition data for 2QnCN, 3QnCN, 3QnB, and 4QnBB, includes vertical excitation energy (Etr, eV), theoretical maximum absorption wavelength (λmax, nm), oscillator strength (OS, *f*), molecular orbital character (MO/character), and primary excitation configuration.

The molecular orbital surfaces reveal a stronger electron density distribution on the CN group in 2QnCN and 3QnCN (Figure 3e,f), confirming its role as an electron-accepting fragment. By contrast, in 3QnB and 4QnBB, the electron density is primarily localized on the oxygen atoms of the benzoxazole moiety (Figure 3g,h).

These findings indicate that the D-π-A design is particularly favorable for 2QnCN and 3QnCN, where efficient intramolecular charge transfer (ICT) is observed. In contrast, the benzoxazole segment in 3QnB and 4QnBB may disrupt this process. Maintaining uninterrupted ICT is crucial as interruptions can result in fluorescence quenching upon interaction with Aβ_1–42._ ^31^ Consequently, 2QnCN and 3QnCN are predicted to be more effective candidates for sustaining fluorescence when binding to Aβ_1–42_.

In the search for efficient fluorophores for detecting Aβ aggregates, 3QnCN stands out as a promising compound due to its D-π-A-π-D architecture, built on a quinoline core with a cyano group, which imparts favorable electronic properties for interaction with amyloid aggregates. This structural configuration is shared by fluorophores such as DADNIR-2 ^51,52^ and FB,^53^ which incorporate dimethylamino groups as electron donors and acceptors, respectively, thereby promoting a strong photophysical response to microenvironmental changes. Similarly, 3QnCN shares with QM-FN-SO_3_ ^52,54^ the aggregation-induced emission (AIE) feature, which is crucial for minimizing background fluorescence and enhancing signals only in the presence of amyloid structures. Furthermore, DBAN probes, such as DBAN-SLM,^52–55^ based on modified cyanine structures, also employ conjugated linkers and terminal donor groups to achieve comparable activation mechanisms.

### Interaction of 2QnCN, 3QnCN, 3QnB, and 4QnBB with a Aβ_1–42_ Fibril through Docking Studies

Molecular docking simulations of quinoline derivatives were performed using an Aβ_1–42_ pentamer (PDB: 2BEG) to identify compounds with stronger affinity for this target. Figure 4a presents the binding energy (ΔG) values for each compound on Aβ_1–42_. The free energy (ΔG) values and binding modes of the ligands were determined on the β-sheet conformation of Aβ_1–42_ as this structure is directly implicated in the aggregation process.^56^ Although Aβ_1–42_ initially assumes an α-helical conformation, it subsequently is converted to a β-sheet structure within the cellular membrane *through* a random coil intermediate, following the catalytic action of γ-secretase.^57^ The compounds showing the most favorable binding energies are 3QnCN and 4QnBB.

**Figure 4:**
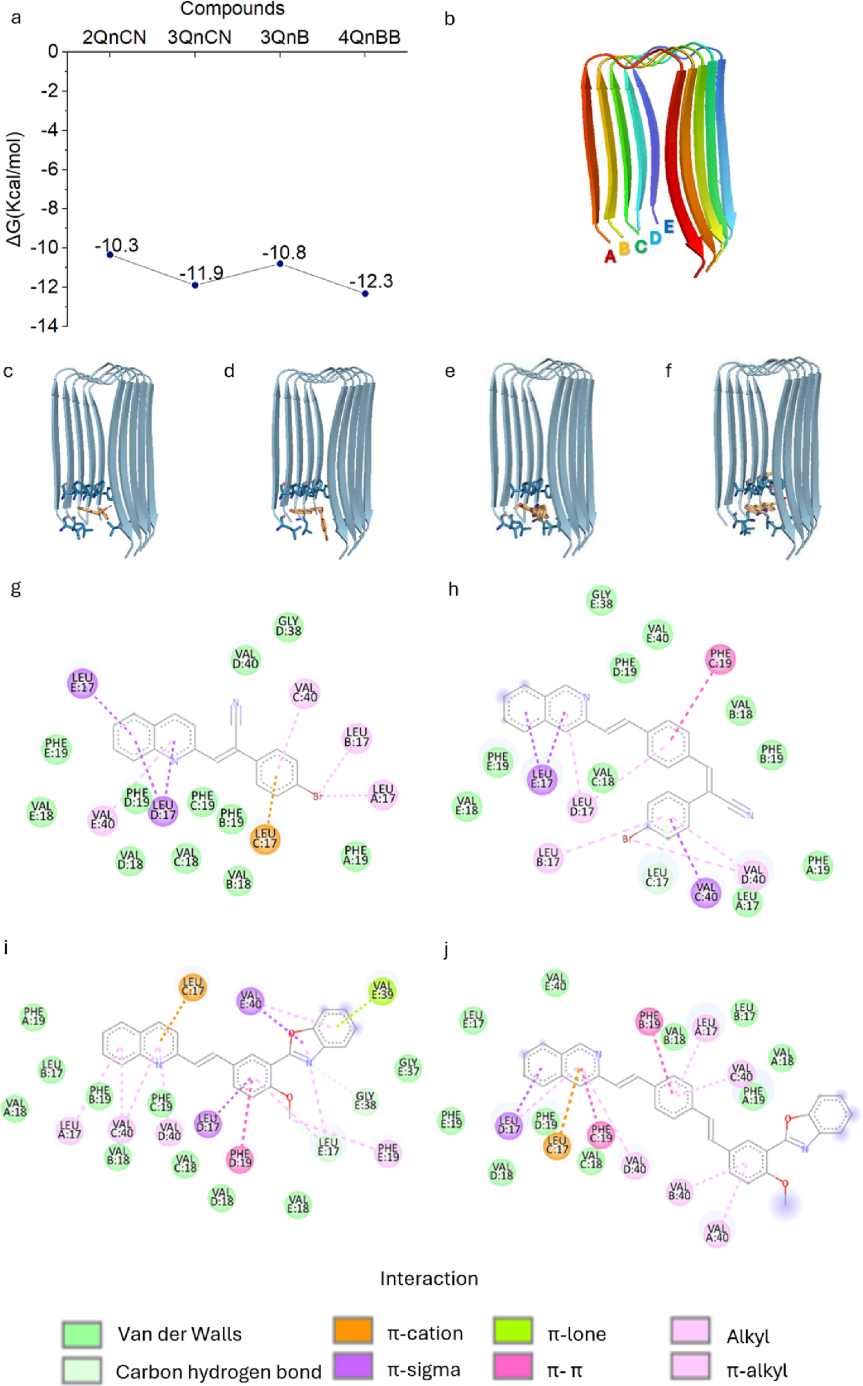
Molecular Docking of Quinoline Derivatives with the Aβ_1–42_ pentamer. (a) Free energy values (ΔG; kcal/mol); (b) PDB: 2BEG, binding site of the pentamer with chains A, B, C, D y E and (c) 2QnCN, (d) 3QnCN, (e) 3QnB, and (f) 4QnBB; interactions of the 2BEG pentamer with (g) 2QnCN, (h) 3QnCN, (i) 3QnB, and (j) 4QnBB. Results were obtained through molecular docking and visualized using Discovery Studio software.

The interactions of the four synthesized compounds with 2BEG (Figure 4b) occur at the entrance of the pentamer cavity, spanning monomers A to E. 2QnCN occupies the entire cavity (Figure 4c). For 3QnCN, carbons 21, 23, and 24 of the quinoline rings extend partly outside the cavity (Figure 4d), whereas for 3QnB, carbons 20, 22, and 23 do so (Figure 4e). For 4QnBB, the benzoxazole segment is positioned externally (Figure 4f).

Compound 2QnCN forms interactions with the amino acid Leu 17 from chain **E** (Leu **E**17) and Leu **D**17 via a π–σ bond on the quinoline rings. It further interacts with Val **E**40 through a π–alkyl bond, with the central phenyl ring via a π–cation interaction with Leu **C**17, and with Val **C**40 through π–alkyl contacts. Finally, residues Leu **A**17 and Leu **B**17 engage in alkyl interactions with the bromine atom (Figure 4g).

Molecular docking studies revealed the following interactions between 3QnCN and the Aβ peptide: π–sigma interactions between Leu **E**17 and the quinoline rings and Leu **D**17 and the central phenyl ring, a π–π stacking interaction between the central benzene ring and Phe **C**19, π–sigma interactions between the bromophenyl group and Val **D**40 residues, and a carbon–hydrogen bond between the benzene ring and Leu **C**17 (Figure 4h).

For 3QnB, the quinoline aromatic rings interact with Leu **A**17, Val **C**40, and Val **D**40 via π–alkyl interactions; with Leu **C**17 through a π–cation bond; with Phe **D**19 via π–π stacking; and with Leu **E**17 through πalkyl contacts. Additionally, the methyl group interacts with Leu **E**17 via a carbon–hydrogen bond and with Phe **E**19 through an alkyl interaction. The benzoxazole segment engages with Leu **E**17 through alkyl contacts, with Val **E**40 via π–sigma and π–alkyl interactions, and with Val **E**39 via a π–lone pair bond. Finally, the nitrogen atom interacts with Gly **E**38 through a carbon–hydrogen bond (Figure 4i).

In 4QnBB, the quinoline ring interacts with Leu **D**17 through π–sigma and π–alkyl contacts, with Leu **C**17 through π–cation, with Phe **C**19 through π–π stacking, and with Val **D**40 through π–alkyl. The central benzene ring establishes π–π stacking with Phe **B**19 and π–alkyl interactions with Leu **A**17 and Val **C**40. Lastly, the phenolic ring interacts with Val **B**40 and Val **A**40 through π–alkyl bonds (Figure 4j).

The compounds 3QnCN and 4QnBB demonstrated more favorable ΔG values when interacting primarily with amino acid residues at site B of Aβ_1–42_. The quinoline rings form π–sigma, π–alkyl, and π–π stack interactions with residues F17 and L17, whereas the central phenyl moiety interacts with F19 and L17. Moreover, in the terminal bromobenzene segment, 3QnCN exhibits π interactions with V40 and L17 at site C. By contrast, interactions of 4QnBB are confined to part of the benzoxazole with V40.

The trimer 3QnCN associates with Aβ_1__–__4__2_, displaying affinity for residues Phe 17, Phe 19, and Val 40, along with enhanced fluorescence and a red-shifted emission. DADNIR-2 ^51,52^ shows similar properties, as do the DBAN probes,^52,55^ which preferentially target soluble Aβ species, engaging in hydrophobic interactions with residues Phe 19 and Val 36. These probes also demonstrate enhanced fluorescence, high biocompatibility, and *in vivo* applicability.

Altogether, 3QnCN integrates structural and functional features characteristic of advanced fluorophores, combining AIE emission efficiency, an optimized electronic framework, and strong specificity toward Aβ, establishing it as a versatile and competitive probe in this field.

### Absorption, Distribution, Metabolism, Excretion (ADME), Toxicological, and Blood–Brain Barrier (BBB) Permeability Prediction

The compounds 2QnCN, 3QnCN, 3QnB, and 4QnBB were evaluated using the SwissADME server to predict physicochemical properties, lipophilicity, hydrophilicity, and pharmacokinetics based on their SMILES codes (Table 3). According to the analysis, 2QnCN and 3QnB comply with Lipinski’s rule of five, whereas 3QnCN and 4QnBB violate the partition coefficient criterion, which governs solubility, adsorption, distribution, and metabolism. Furthermore, 4QnBB shows a molar reactivity value exceeding 130, suggesting higher overall polarity. Toxicity parameters, including LD50 values, carcinogenicity, immunotoxicity, mutagenicity, and cytotoxicity, were predicted using the ProTox-II platform (Table 3). Results indicate that 3QnB and 4QnBB conform to class 5 toxicity, whereas 2QnCN and 3QnCN exhibit LD50 values of 1760 and 1000 mg/kg, respectively. Carcinogenicity is predicted to be active for 3QnB and 4QnBB; immunotoxicity for 3QnCN, 3QnB, and 4QnBB; and mutagenicity for all compounds. None of the compounds is predicted to be inactive for cytotoxicity. Finally, BBB permeability is predicted to be feasible only for 2QnCN.

**Table 3:** Absorption, Distribution, Metabolism, Excretion (ADME), Toxicological, and Permeability Predictions of 2QnCN, 3QnCN, 3QnB, and 4QnBB^a^

molecule	MW (g/mol)	heavy atoms	aromatic heavy atoms	fraction Csp3	rotable bonds	H bond donors	MR	TPSA (A°^2^)	Lipinski violations	permeability BBB
2QnCN	335.20	21	16	0.00	2	2	89.37	36.68	0	Si
3QnCN	437.33	29	22	0.00	4	2	124.74	36.68	1: MLOGP > 4.15	no
3QnB	378.42	29	25	0.04	4	4	116.61	48.15	0	no
4QnBB	480.56	37	31	0.03	6	0	151.98	48.15	1: MLOGP > 4.15	no

predicted toxicity										
			**carcinogenicity**		**immunotoxicity**		**mutagenicity**		**cytotoxicity**	
molecule	**class**	**LD50** (mg/kg)	prediction	probability	prediction	probability	prediction	probability	prediction	probability
2QnCN	4	1760	inactive	0.78	inactive	0.85	active	0.69	inactive	0.69
3QnCN	4	1000	inactive	0.78	active	0.56	active	0.69	inactive	0.63
3QnB	5	3000	active	0.53	active	0.95	active	0.56	inactive	0.82
4QnBB	5	3000	active	0.53	active	0.98	active	0.56	inactive	0.82

a Fraction Csp3: fraction of sp3 carbons, MR: molar refractivity, TPSA: topological polar surface area.

Although 3QnCN, 3QnB, and 4QnBB do not cross the BBB, and despite 4QnBB showing a more favorable ΔG, theoretical calculations indicate that ICT is disrupted within the benzothiazole ring, as observed in 3QnB. In contrast, while 2QnCN is the only compound predicted to cross the BBB, 3QnCN demonstrates superior optical properties compared to 2QnCN. Moreover, carcinogenicity and cytotoxicity predictions suggest that 3QnB and 4QnBB are active, whereas 3QnCN remains inactive, supporting the rationale for conducting *in vitro* studies with 3QnCN.

### 3QnCN Cytotoxicity and Fluorescence Determination in PC12 Cells

The PC12 model was employed because Aβ_1–42_ has been reported to interact with unfixed rat pheochromocytoma PC12 cells, a process that can be visualized using the amyloid-specific dye CR. Aβ_1–42_ preferentially binds to ganglioside- and cholesterol-rich membrane domains, forming amyloids in a time-dependent manner.^58^ Furthermore, PC12 cells express receptors directly implicated in cognitive processes that are altered in AD.^59^

Therefore, the addition of Aβ_1–42_ to PC12 cells to induce aggregate formation is of particular importance because these aggregates can be detected by fluorescent compounds. In addition, control PC12 cells form fewer amyloids than transfected cells that overexpress the amyloid peptide. This difference arises because amyloid beta is associated with the junctional apparatus and may contribute to increased intercellular adhesion. Likewise, the cell surface displays structural and functional alterations that promote aggregate formation.^60^

Before to assays the staining Aβ_1–42_ aggregates with the compound in the PC12 cells the cytotoxicity assays were done using the MTT method revealed that 3QnCN is cytotoxic to PC12 cells at a concentration of 100 μM, causing death in approximately 31% of cells (Figure 5a). In contrast, cell viability remains high (above 83%) at concentrations of 50 μM or lower. Fluorescence intensity was quantified using a plate assay (Figure 5b), and the results confirmed that fluorescence can be reliably measured in cell cultures. At a concentration of 6.25 μM, fluorescence intensity increased by 33%, followed by 43% at 12.5 μM, 62% at 25 μM, 66% at 50 μM, and 88% at 100 μM, compared with the negative control lacking 3QnCN. The positive control was defined as treatment with 100 μM 3QnCN in the absence of Aβ_1–42_, which exhibited a fluorescence intensity of 100%.

**Figure 5:**
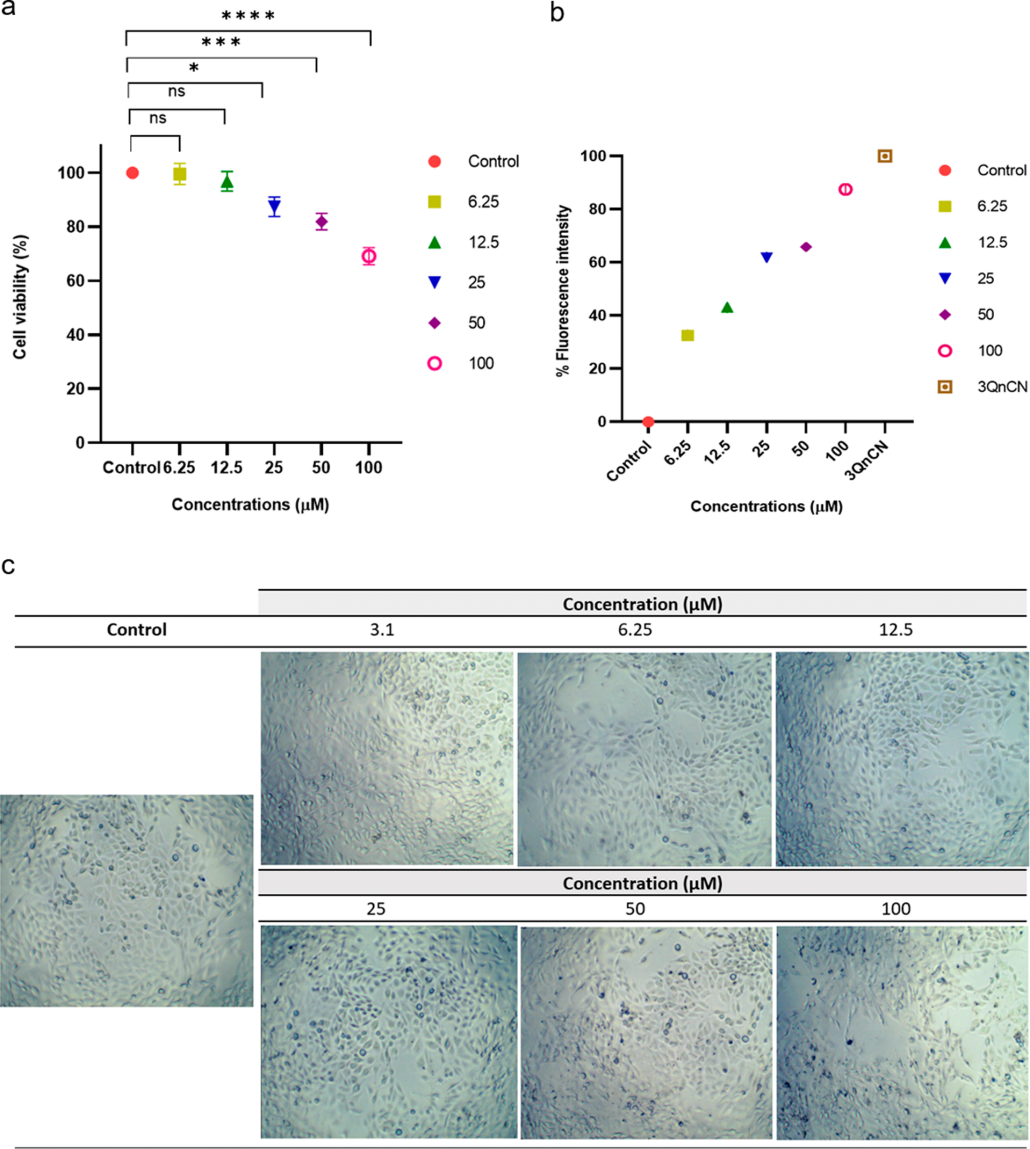
Cytotoxic effects of 3QnCN at 48 h in the PC12 cell line. (a) Cell viability assessed by MTT assay; (b) quantification of fluorescence in cells; (c) PC12 morphology after treatment with 3QnCN at 3.1, 6.2, 12, 25, and 100 μM for 48 h at 4× magnification. Graphs show mean ± SEM (*p < 0.05, **p < 0.001, and ***p < 0.0001), determined using Dunnett’s multiple comparisons test between concentrations and control cells (medium plus 0.02% DMSO–DMF).

Figure 5c shows the characteristic star-shaped morphology of PC12 cells in the control group. This morphology was preserved at concentrations 50 μM, but at 100 μM, cell death was evident, accompanied by precipitation of 3QnCN after 48 h of incubation at 37 °C.

The trimer 3QnCN shows a violation of Log P according to Lipinski’s rule of five, reflecting limitations in solubility. Nevertheless, previous studies have reported successful *in vitro* assays using DMSO as a solvent.^8^ The nontoxicity of 3QnCN was confirmed by *in vitro* assays, showing that cell viability remained above 80% even at concentrations of 50 μM. With respect to BBB permeability, three of the four compounds are predicted to be unable to cross. However, prior reports on related compounds have yielded favorable outcomes. For example, CRANAD-3, a marker recently evaluated *in vivo,* successfully visualized amyloid plaques in transgenic mice despite predictions of poor BBB penetration. Images of deep cortical layers confirmed its efficacy, and the administered doses did not induce microglial inflammation in either healthy brains or AD mice.^61^ Similarly, Xu et al. developed two quinoline–malononitrile-based markers, with molecular weights exceeding 500 g/mol and having good water solubility, specifically designed for detecting and imaging Aβ aggregates. *In vitro* studies showed that these markers displayed high affinity for Aβ aggregates, enhancing fluorescence through ICT. Although these probes were non-BBB-permeable, they successfully visualized Aβ plaques in brain sections of transgenic Alzheimer’s mice.^49^ Therefore, despite predictions regarding BBB permeability, 3QnCN emerges as a promising candidate for *in vivo* studies.

### Fluorescence Assay to Evaluate the Interaction of 3QnCN and Aβ_1–42_ Aggregation

Given that aggregation-induced compounds can emit fluorescence upon interacting with Aβ, 3QnCN was subjected to a fluorescence assay in culture. After stimulating the cells with Aβ_1–42_, fluorescence was successfully quantified under the established parameters. The interaction between 3QnCN and Aβ_1–42_ was evaluated using a fluorescence assay (Figure 6). Following 48 h of incubation of Aβ_1–42_ + 3QnCN complexes, fluorescence was measured at concentrations of 1X, 2X, 3X, and 4X, where 1X corresponds to 22 μM Aβ_1–42_ + 0.06 μM 3QnCN. The emission spectra revealed an increase in fluorescence intensity after 48 h at different complex concentrations. Since 3QnCN displays a maximum emission peak at 439 nm, corresponding to π–π* electronic transitions in the blue region of the spectrum, a red shift of 126 nm was observed upon complex formation. At the 1X concentration, two peaks were detected at 436 and 463 nm, with the first diminishing at higher concentrations. The band at 463 nm is attributed to complex formation, which enhances π–π* transitions located in the green region of the spectrum. Additionally, the fibrillar state of Aβ_1–42_ was confirmed by a ThT assay and further supported by atomic force microscopy (Figure S1).

**Figure 6:**
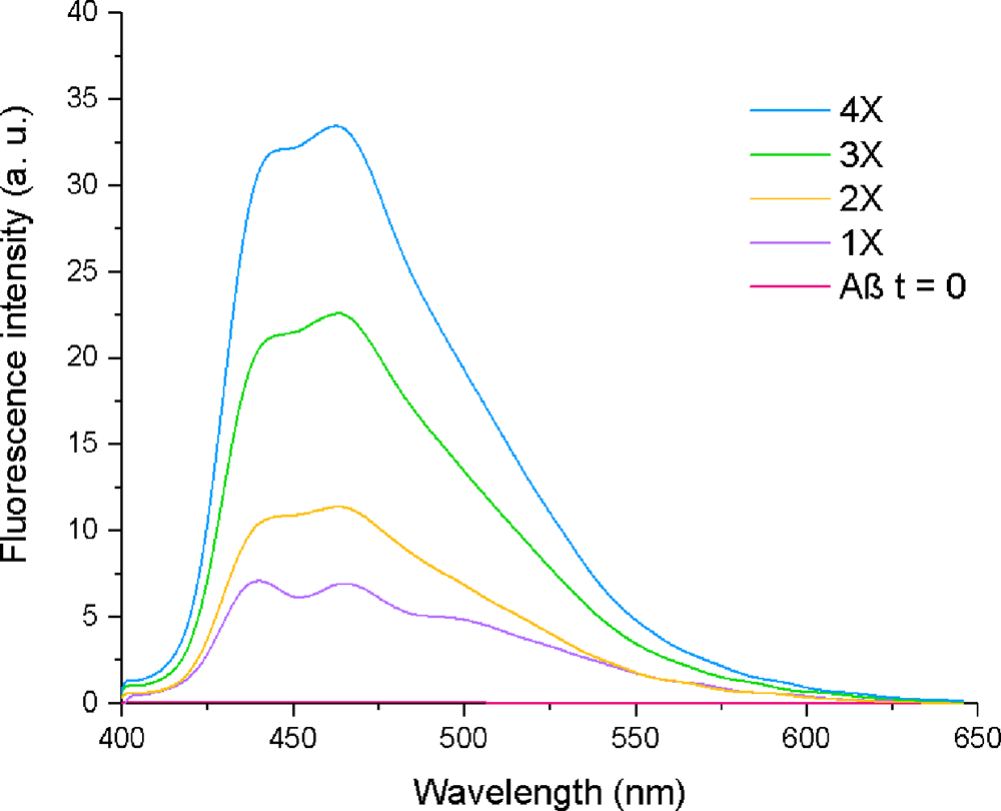
Interaction of 3QnCN in Milli-Q water and dimethyl sulfoxide (DMSO) < 0.1% with Aβ_1–42_ 1X = 22 μM Aβ_1–42_ + 0.06 μM 3QnCN, 2X = 44 μM Aβ_1–42_ + 0.12 μM 3QnCN, 3X = 66 μM Aβ_1–42_ + 0.18 μM 3QnCN, 4X = 88 μM Aβ_1–42_ + 0.24 μM 3QnCN.

Monitoring of fluorescence intensity in the Aβ_1–42_ + 3QnCN complex reveals a shift to longer wavelengths accompanied by an increase in the fluorescence intensity. This behavior parallels previous findings that showed marked changes in the optical properties of quinoline derivatives, where variations in the number and position of nitrogen atoms produced higher emission maximum, increased quantum yields, and strong affinity for synthetic Aβ_1–42_ upon interaction.^35^

### Time- and Dose-Dependent Fluorescent Labeling of Aβ_1–42_ in PC12 Cells Using 3QnCN

Images were captured using confocal microscopy on PC12 cells stimulated with Aβ_1–42_ and treated with different concentrations of 3QnCN for varying time points: 25 μM and 50 μM for 24 h, 50 μM for 1 h, and 0.05% for 30 min. ThS was used as a positive control. As shown in Figure 7, cells treated only with Aβ_1–42_ did not exhibit fluorescence in any of the assays. However, when cells were stimulated with Aβ_1–42_ and treated with 3QnCN at 25 or 50 μM, fluorescence intensity increased compared with Aβ_1–42–only cells_ and cells treated with 3QnCN alone without stimulation. In the latter case, the fluorescence observed was minimal and attributed to compound aggregates, causing nonspecific labeling. The fluorescence signal localized around the nucleus, showing greater intensity at 50 μM, whereas the cell morphology remained intact. By contrast, ThS treatment caused abnormal cell morphology, which hindered signal quantification due to overexposure; ThS penetrated the cytoplasm and nucleus, likely as a consequence of the prolonged 24-h treatment (Figure 7b).

**Figure 7:**
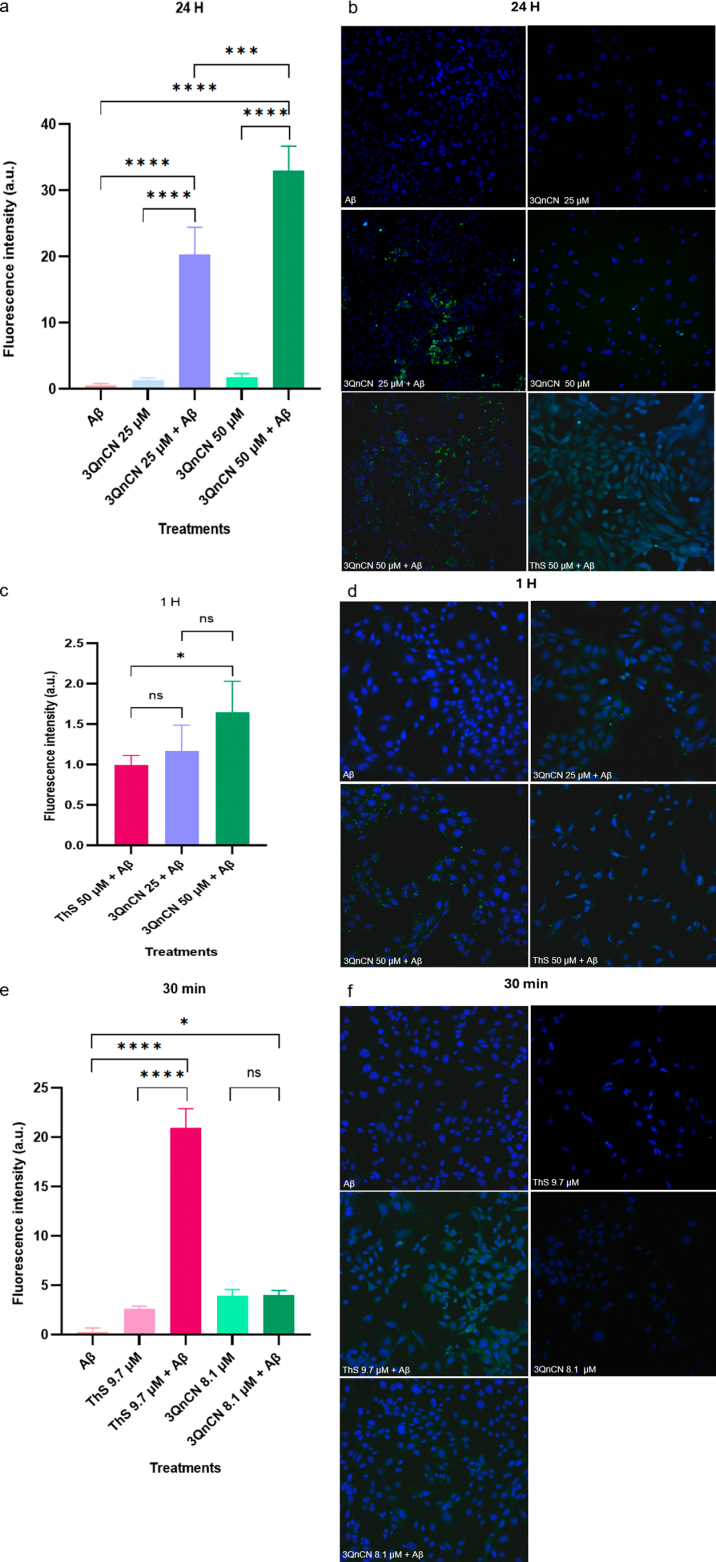
Comparative microscopy and quantitative analysis of 3QnCN and ThS in Aβ_1–42‑_stimulated PC12 cells: (a) statistical analysis after 24 h treatment; (b) 40× micrographs of control Aβ, 3QnCN 25 μM, 3QnCN 25 μM + Aβ, 3QnCN 50 μM, 3QnCN 50 μM + Aβ, and ThS 50 μM + Aβ; (c) statistical analysis after 1 h treatment; (d) 40× micrographs of the control Aβ, 3QnCN 25 μM + Aβ, 3QnCN 50 μM + Aβ and ThS 50 μM + Aβ; (e) statistical analysis after 30 min treatment; (f) 40× micrographs of control Aβ, ThS 9.7 μM, ThS 9.7 μM + Aβ, 3QnCN 8.1 μM and 3QnCN 8.1 μM + Aβ. Each point represents the mean ± SEM (*p < 0.05, **p < 0.001, and ***p < 0.0001) Tukey’s multiple comparisons test.

In the 1-h experiment, no marked difference was observed between ThS at 50 μM and 3QnCN at 25 μM, unlike the comparison between ThS at 50 μM and 3QnCN at 50 μM. A clear distinction was noted between the effects of 3QnCN at 25 and 50 μM (Figure 7c). Treatments with 3QnCN at both concentrations showed normal cell growth with fluorescence signals concentrated around the nucleus, with stronger intensity at 50 μM. In contrast, while ThS produced a detectable signal, the cells displayed reduced confluence and elongated morphology, likely reflecting stress induced by the concentration, similar to the results of the 24-h assay (Figure 7d).

When the treatment time was reduced to 30 min at concentrations of 3QnCN (8.1 μM) and ThS (9.7 μM), corresponding to 0.05% w/v as previously reported for labeling PC12 cells,^62^ 3QnCN treatment produced low fluorescence, with no remarkable difference compared to untreated cells. In contrast, a marked increase in the signal intensity was observed for the ThS-positive control compared with 3QnCN (Figure 7e). Both treatments resulted in uniform cell growth (Figure 7f). These results suggest that the quinoline trimer requires at least 1 h to establish an effective interaction with Aβ_1–42_ and efficiently label it.

The percentage of labeled cells relative to the total cell count was also analyzed following treatments with 3QnCN and ThS. With 3QnCN at 25 and 50 μM for 24 h, 80% and 98% of cells were labeled, respectively. Under 1 h treatments, labeling rates were 35% (25 μM) and 84% (50 μM) for 3QnCN and 29% for ThS. After 30 min, 3QnCN labeled 10% of cells, whereas ThS labeled 98% (Figure S2).

3QnCN 8.1 μM + Aβ

The compound 3QnCN shows high specificity for Aβ_1–42_, without interference from other biomolecules or alteration in the cell morphology, making it highly selective for *in vitro* amyloid plaque studies. Similar selectivity is reported for QM-FN-SO_3_,^52,54^ which is insensitive to Aβ monomers, amino acids, and metabolites, thereby reducing the likelihood of false positives. In contrast, DADNIR-2 ^51,52^ detects fibrillar Aβ and tau aggregates, whereas 3QnCN targets Aβ1–42 exclusively. Compared to DBAN-SLM,^52,55^ which binds monomeric and oligomeric forms, 3QnCN specifically labels the fibrillar form of Aβ.

Regarding biocompatibility, 3QnCN demonstrates low cytotoxicity, a property shared with QM-FN-SO_3_,^52,54^ DADNIR-2,^51,52^ and DBAN, all of which exhibit low toxicity, without inducing autofluorescence or cellular alterations.

The trimer 3QnCN responds to hydrophobic environments with increased fluorescence intensity and red-shifted emission, a behavior also seen in QM-FN-SO_3_,^52,54^ DADNIR-2,^51,52^ and DBAN. This spectral sensitivity supports its strong performance in confocal microscopy, where 3QnCN produces a clear perinuclear signal after 1 and 24 h of treatment without altering the cell morphology. Although undetectable at low concentrations and short incubation durations, no cytotoxic effects were observed at higher concentrations. This response resembles the selective activation of QM-FN-SO_3_ ^52,54^ and the *in vivo* versatility of DADNIR-2 ^51,52^ and DBAN.^52,55^

For the signal-to-noise ratio (S/N), 3QnCN performs remarkably, with low background fluorescence, similar to QM-FN-SO_3_.^52,54^ DADNIR-2 ^51,52^ and DBAN^52,55^ achieve signal enhancements of up to 70- and 126 fold, respectively. Overall, 3QnCN shares with these probes key features—molecular architecture, binding mechanism, low cytotoxicity, responsive behavior to Aβ, and high specificity—yet clearly outperform conventional markers like ThS. These properties underscore its promise for Aβ_1–42_ labeling and potential *in vivo* applications.

Unlike 3QnB and 4QnBB, in which the benzoxazole unit disrupts ICT, 3QnCN preserves continuous ICT through its cyano group and quinoline conjugated system, yielding greater photophysical efficiency and stable emission. It has a higher quantum yield than 2QnCN, 3QnB, and 4QnBB, resulting in more intense fluorescence and shows a smaller Stokes shift, indicating reduced energy loss from nonradiative processes. Molecular docking further reveals 3QnCN binding to key residues (Phe17, Phe19, Val40, and Leu17), forming stable π–π and π–alkyl interactions. These interactions enhance its selective binding to the fibrillar form of Aβ while minimizing interference from other biomolecules. Like advanced fluorophores such as QM-FN-SO_3_, 3QnCN enhances fluorescence only in the presence of Aβ aggregates, thereby lowering background noise and improving the signal-to-noise ratio. This property is less defined in 2QnCN, 3QnB, and 4QnBB.

Cell viability assays confirm that 3QnCN maintains >80% viability up to 100 μM, indicating low cytotoxicity. It matches leading fluorophores (DADNIR-2, QM-FN-SO_3_, and DBAN) but offers greater specificity for Aβ_1–42_. Unlike DADNIR-2, which also binds tau aggregates, 3QnCN selectively targets Aβ_1–42_, reducing the possibility of false positives and making it well-suited for amyloid plaque detection. Compared with DBAN-SLM, which recognizes monomeric and oligomeric forms, 3QnCN focuses on fibrils—critical for detecting AD plaques.

In PC12 cells, 3QnCN produces a distinct perinuclear signal after 1 and 24 h of treatment without altering the cell morphology. It shows strong fluorescence sensitivity to hydrophobic environments, with red-shifted emission and signal amplification, comparable to state-of-the-art fluorophores but with lower autofluorescence.

## Materials and Methods

### Synthesis of 2QnCN, 3QnCN, 3QnB, and 4QnBB

Reagents were obtained from Sigma-Aldrich and solvents from J.T. Baker, and all were used per supplier protocols. The synthetic route is shown in Figure 1.

### Synthesis of 3QnCN

#### (*E*)-4-(2-(Isoquinolin-3-yl)­vinyl)­benzaldehyde (2QnB)

In a 50 mL round-bottomed flask, 0.05 g (0.34 mmol) of 3-methylisoquinoline (99%), 0.046 g (0.34 mmol) of terephthaldehyde (99%), and 3 mL of Ac_2_O were combined. The mixture was stirred and refluxed for 24 h at 120 °C under N_2_. Progress was monitored by thin-layer chromatography (TLC) on alumina plates (Al_2_O_3_). Ice was then added, and the mixture was stirred for 24 h. The product was washed sequentially with cold CH_3_OH, then cold water, and dried, yielding a yellow powder (0.087 g, 97%).

#### (*E*)-2-(4-Bromophenyl)-3-(4-((E)-2-(isoquinolin-3-yl)­vinyl)­phenyl)­acrylonitrile (3QnCN)

In a 50 mL flask, 0.03 g (0.11 mmol) of 2QnB and 0.023 g (0.12 mmol) of 2-(4-bromophenyl)­acetonitrile (99%) were combined with 4 mL of 5% KOH in CH_3_OH. The mixture was stirred for 8 h, then filtered, washed with cold water and methanol, vacuum-filtered, and dried. A yellow powder was obtained (0.049 g, 97%). UV–Vis (DMSO): λmax 378 nm. Fluorescence 439 nm. IR (ATR cm^–1^): ν­(CH aromatic) 3054 ν­(CH aliphatic) 2925, 2852 ν­(CN) 2210 ν­(C = C aromatic) 1736 ν­(C = N) 1594 ν­(C = C aliphatic) 1493 (Figure S3). ^1^H NMR (25 °C, CDCl_3_, 400 MHz δ ppm: 8.1 (m, 3H, quinoline), 7.9 (m, 2H, J = 6.8 Hz, quinoline, phenyl, and vinyl), 7.7 (m, 6H, quinoline, phenyl, and vinyl), 7.4 (m, 5H, phenyl) (Figure S4a). ^13^C NMR: δ 113.1 (C-8), 117.9 (C-28), 119.5 (C-18), 126.4 (C-24), 127.3 (C-13, C-9), 127.4 (C-6, C-1), 127.7 (C-14, C-11, C19), 129.9 (C-3, C-5, C-22, C29), 130.2 (C-10), 130.5 (C-23-C25), 130.9 (C-20), 132.42 (C-4), 133.4 (C-21), 133.1 (C-12), 136.5 (C-2), 141.7 (C-7), 148.3 (C-17), 155.3 (C-15) (Figure S4b). MS: *m*/*z* 437 (Figure S8).

### Synthesis of 2QnCN

#### (*Z*)-2-(4-Bromophenyl)-3-(quinolin-2-yl)­acrylonitrile (2QnCN)

In a 50 mL flask, 0.05 g (0.32 mmol) of quinoline-2-carbaldehyde (99%) and 0.075 g (0.38 mmol) of 2-(4-bromophenyl)­acetonitrile (99%) were dissolved in 5 mL of 5% KOH in CH_3_OH. The mixture was stirred for 8 h, then filtered, washed with cold water, vacuum-filtered, washed with cold methanol, and dried. A white powder was obtained (0.10 g, 95%). UV–Vis (DMSO) λmax 366 nm. Fluorescence 329 nm. IR (ATR cm^–1)^: ν­(CH aromatic) 3054 ν­(CH aliphatic) 2925 2852 ν­(CN) 2222 ν­(C = C aromatic) 1736 ν­(C = N) 1594 ν­(C = C aliphatic) 1493 (Figure S3). ^1^H NMR (25 °C, CDCl_3_, 400 MHz) δ ppm: 8.2 (m, 3H, quinoline), 7.8 (m, 5H, quinoline/phenyl), 7.7 (m, 2H, quinoline, phenyl, and vinyl) (Figure S5a). ^13^C NMR: δ 115.0 (C-19), 119.3 (C-20), 120.8 (C-9), 127.1 (C-15), 127.2 (C-1), 127.6 (C-5), 129.9 (C-13, C-17), 127.9 (C-6), 130.2 (C-3), 130.4 (C-2), 132.8 (C-14, C-16), 136.4 (C-12), 137.0 (C-10), 141.6 (C-11), 148.1 (C-4), 153.0 (C-8) (Figure S5b). MS: *m*/*z* 337 (Figure S9).

### Synthesis of 4QnBB

#### (*E*)-3-(4-Vinylestyril)­isoquinoline (**6**)

In a 50 mL round-bottomed flask, 0.05 g (0.019 mmol) of 2QnB and 0.068 g (0.019 mmol) of MTPP-Br were dissolved in 20 mL THF. The mixture was subjected to three cycles of vacuum – N_2_ (10 s each) and was then stirred under N_2_ in an ice bath for 15 min. Subsequently, 0.021 g (0.019 mmol) of *t*-BuOK was added, and the mixture was stirred at 20–25 °C for 24 h under N_2_. Progress was monitored by TLC (Al_2_O_3_ plates). The product was purified by column chromatography on alumina using hexane: ethyl acetate (9:1) as eluent, yielding a yellow powder.

#### 2-(5-((*E*)-4-((*E*)-2-(Isoquinolin-3-yl)­vinyl)­styryl)-2-methoxyphenyl)­benzo­[d]­oxazole (4QBB)

In a 250 mL flask, 0.047 g (0.15 mmol) of 2Ben and 0.020 g (0.12 mmol) of compound 6 were combined with 7.5 mg POT (99%) and 14 mg Pd­(OAC)_2 (99%)_. Then, 50 mL of anhydrous Et_3_N: DMF (80:20) was added, and three vacuum–N_2_ cycles (10 s each) were performed. The mixture was refluxed at 120 °C for 48 h. The product was filtered, washed with CH_2_Cl_2_:water, concentrated, and precipitated dropwise into cold CH_3_OH. The solid was centrifuged and dried, yielding a brown powder (0.017 g, 47%). UV–Vis (DMSO): λmax 396 nm. Fluorescence 389 nm. IR (ATR cm^–1^): ν­(CH aromatic) 3054 ν­(CH aliphatic) 2925, 2852 ν­(C = C aromatic) 1704 ν­(C = N) 1594 ν­(C = C aliphatic) 1452 ν­(C–O aliphatic) 1268 (Figure S3). ^1^H (25 °C, CDCl_3_, 400 MHz, δ ppm): 7.7 (m, 2H, quinoline), 7.5 (m, 3H, quinoline/phenyl), 7.2 (m, 7H, J = 6.8 Hz, quinoline, phenyl, and vinyl) 6.9 (m, 7H, J = 6.8 Hz, phenyl and vinyl) 3.9 (m, 3H, CH_3_) (Figure S6a). ^13^C NMR: δ 56.54 (C-8), 110.5 (C-9), 112.8 (C-3), 113.9 (C-5), 117.9 (C-35), 120. (C-12), 121.0 (C-13), 126.5 (C-14), 126.6 (C-36), 125.3 (C-19, C-21,C-23, C-24), 128.4 (C-30), 128.6 (C-26), 129.6 (C-31), 129.6 (C-31, C-28), 131.0 (C-6), 132.0 (C-27), 131.1 (C-2), 131.9 (C-1), 136.5 (C-17), 133.6 (C-37), 135.2 (C-29), 135.6 (C-20), 137.0 (C-22), 147.9 (C11), 150.3 (C-10), 150.9 (C-34), 154.6 (C-32), 157.5 (C-4), 160.0 (C-7) (Figure S6b).MS: *m*/*z* 379 (Figure S10).

### Synthesis of 3QnB

#### 5-Bromo-2-hydroxybenzaldehyde (**9**)^45^

The synthesis was carried out following López-Ruiz et al. (2011). In a 50 mL flask, 5-bromo-2-hydroxybenzaldehyde (0.95 g, 4.60 mmol) and 2-aminophenol (0.50 g, 4.60 mmol), both 99% pure (Sigma-Aldrich), were reacted with NaCN and phenylboronic acid in methanol at room temperature for 4 h. The product was precipitated with sodium acetate, affording a pink powder (mp = 164 °C).

#### 2-(5-Bromo-2-methoxyphenyl)­benzo­[d]­oxazole (2Ben)^45^

Following López-Ruiz et al. (2011), 0.05 g (0.17 mmol) of compound 9, 0.42 g (1.7 mmol) of CH_3_I (99% Sigma-Aldrich), and K_2_CO_3_ (99%, Sigma-Aldrich) were refluxed in acetone for 18 h. The reaction mixture was filtered with cold CH_3_OH, yielding an orange powder (95%.

#### 2-Vinylquinoline (**5**)

In a 50 mL flask, 0.20 g (1.27 mmol) of 2-quinolinecarbaldehyde (99%) and 0.50 g (1.39 mmol) of methyltriphenylphosphonium bromide (MTPP-Br 99%) were dissolved in 20 mL tetrahydrofuran (THF). The solution underwent three vacuum–N_2_ cycles (10 s each) and was stirred under N_2_ in an ice bath for 15 min. Then, 0.15 g (1.34 mmol) of *t*-BuOK (99%) was added, and the reaction mixture was stirred at room temperature for 24 h under N_2_. Progress was monitored by TLC (Al_2_O_3_ plates). The reaction mixture was purified with column chromatography on alumina with hexane:ethyl acetate (9:1) as the eluent, affording the product as a brown oil.

#### 2-(5-((*E*)-4-((*E*)-2-(Isoquinolin-3-yl)­vinyl)­styryl)-2-methoxyphenyl)­benzo­[d]­oxazole (3QnB)

In a 250 mL flask, 0.020 g (0.012 mmol) of compound 5 and 0.040 g (0.016 mmol) of 2Ben were combined with 7.5 mg POT (99%) and 14 mg Pd­(OAC)_2_ (99%). Then, 50 mL of anhydrous Et_3_N:DMF (80:20) was added, followed by three vacuum–N_2_ cycles (10 s each). The mixture was refluxed at 120 °C for 48 h. The product was filtered, washed with CH_2_Cl_2_water, concentrated, and precipitated dropwise into cold CH_3_OH. The solid was centrifuged and dried, affording a brown powder (0.029 g, 62%).

UV–Vis (DMSO): λmax 363 nm. Fluorescence 374 nm. IR (ATR cm^–1)^: ν­(CH aromatic) 3054 ν­(CH aliphatic) 2925, 2852 ν­(C = C aromatic) 1718 ν­(C = N) 1594 ν­(C = C aliphatic) 1452 ν­(C–O aliphatic) 1268 (Figure S3)^1^H (25 °C, CDCl_3_, 400 MHz δ ppm): 7.9 (m, 3H, quinoline), 7.8 (m, 4H, quinoline/phenyl), 7.4 (m, 4H, J = 6.8 Hz, quinoline, phenyl, and vinyl) 7.1 (m, 3H, phenyl), 3.8 (m, 3H, CH_3_) (Figure S7a). ^13^C NMR: δ 56.1 (C-18), 110.5 (C-9), 111.7 (C-3), 117.3 (C-5), 120.1 (C-12), 119.1 (C-27), 124.5 (C-13), 124.3 (C-14), 125.5 (C-19), 131.8 (C-23), 128.5 (C-1, C-21), 132.0 (C-29), 127.8 (C-24), 129.2 (C-6), 135.8 (C-28), 132.1 (C-20), 132.9 (C-2), 133.7 (C-17), 138.9 (C-11), 138.9 (C-11), 143.5 (C-22), 148.2 (C-10), 150.6 (C-26), 155.8 (C-4), 160.4 (C-7). (Figure S7b). MS: *m*/*z* 449 (Figure S11).

### Chemical Characterization

^1^H and ^13^C NMR spectra were recorded on an Agilent Varian (400 MHz) instrument using deuterated chloroform (CDCl_3_) with tetramethylsilane (TMS) as the internal reference. Infrared spectra were obtained by ATR-FTIR using a PerkinElmer Frontier spectrophotometer. Electrospray ionization mass spectra (ESI-MS) were acquired on a microTOF-Q instrument (method: tune positive low 01.m).

### Optical Characterization

UV–Vis absorption spectra were recorded in spectroscopic-grade DMSO at room temperature using a PerkinElmer Lambda XLS spectrophotometer over 300–900 nm. Fluorescence spectra were measured with a PerkinElmer LS55 spectrophotometer using standard 1 cm quartz cells and spectroscopic-grade DMSO as the solvent.

### *In* *Silico* Evaluation

#### Preparation and Optimization of Ligands for Docking Studies

2D representations of the compounds were generated using ACD/ChemSketch 14.01 software (Toronto, ON, Canada).^63^ The structures were then preoptimized by adding hydrogen atoms and converting them to 3D format for storage as *.mol files. A Z-matrix for each ligand was prepared with the GaussView 5.0.8 program. Energy minimization was carried out using the semiempirical AM1 method. The three-dimensional integrity of the minimized structures was confirmed, and a *.pdb file was created. Finally, the structures were further optimized with the Avogadro program to generate the *.pdb file used for docking simulations.

#### Preoptimization of the Protein for Docking Studies

For docking analysis, the β-sheet conformation of Aβ_1–42_ (PDB: 2BEG) was used after manually removing water molecules in a text editor. Gasteiger charges, polar hydrogens, and Kollman charges were added, and the *.pdbqt file was generated with AutoDock Tools 1.5.6.^63^

#### Docking Studies

For docking studies, proteins were kept rigid, whereas ligands were treated as flexible. The *.pdb, *.pdbqt, *.gpf, and *.dpf files were generated in AutoDock Tools. Following docking simulations, protein–ligand interactions were examined using the same software. The grid box measured 126 Å^3^ and was centered on the protein. Docking employed the Lamarckian genetic algorithm in AutoDock Tools, with an initial population of 500 and 1 × 10^7^ evaluations. Ligand–protein complexes were then assessed for the lowest free energies (ΔG) to characterize binding interactions.

#### Visualization of Protein–Ligand Interactions

Ligand–protein interactions were visualized using Py 2.5.2 ^64^ and BIOVIA Discovery Studio Visualizer 4.5.0 ^65^

### Theoretical Calculations

Computational calculations were performed with Gaussian 09, and graphs were generated with the GaussView 5.1 interface.^66,67^ Ground-state geometry of 2QnCN, 3QnCN, 3QnB, and 4QnBB was optimized using the hybrid exchange–correlation functional B3LYP with the 6–31G (d,p) basis set. HOMO, LUMO, and band gap energies were obtained from the most stable optimized structures, and electron distributions were visualized in GaussView 5.1. Electronic excitations were calculated by TD-DFT at the BhandHLYP/6–31G­(d,p) level.^68^

### ADME, Toxicological, and BHE Permeability Prediction

Physicochemical properties were predicted using SwissADME.^69^ whereas toxicity profiles were evaluated with the ProTox-II server^70^ based on the Globally Harmonized System of Classification and Labeling of Chemicals (GHS) categories.

### *In* *Vitro* Assays

#### Evaluation of Aβ_1–42_ Labeling *In Vitro* by Fluorescence Assay

The evaluation of 3QnCN as a marker for Aβ_1–42_ fibril formation was performed as follows: A solution of Aβ_1–42_ (Calbiochem, cat. no. PP69) at 0.25 μg/μL in Milli-Q water was incubated with or without 100 μM 3QnCN (DMSO < 0.1%) in a quartz cell at 37 °C under continuous stirring for 48 h. Aliquots were collected at concentrations of 1X (22 μM Aβ_1–42_ + 0.06 μM 3QnCN), 2X, 3X, and 4X. Fluorescence increase was recorded at an excitation wavelength of λ = 368 nm, with a slit width of 15.0 for excitation and 3.0 nm for emission, over the range of 388 to 650 nm. Fluorescence measurements were performed on an LS-55 spectrofluorometer (PerkinElmer).

#### Cytotoxic Evaluation of Compounds in PC12 Cells

PC12 cells were cultured in Dulbecco’s modified Eagle’s medium supplemented with 10% fetal bovine serum and 1% antibiotic–antimycotic (1% penicillin G, sodium salt, and streptomycin sulfate) 37 °C in a 5% CO_2_ atmosphere. Cell handling and visualization were performed in a biosafety level 2 vertical laminar flow cabinet and an inverted binocular microscope, respectively. Cells were detached using 20% trypsin in PBS and seeded in 96-well plates at 1 × 10^4^ cells/well. After 24 h, the medium was replaced with one of the following treatments: medium alone, medium with 0.02% DMSO–DMF, or medium with 3QnCN (6.25, 12, 25, 50, or 100 μM), followed by 24-h incubation. For viability testing, 3-(4,5-dimethylthiazol-2-yl)-2,5-diphenyltetrazolium bromide (MTT) was used. At the end of the incubation, the medium was replaced with 50 μL MTT solution (0.5 mg/mL PBS) and incubated for 4 h under standard culture conditions. The MTT solution was then removed, and 50 μL DMSO was added to dissolve the formazan crystals. The absorbance was recorded at 550 nm using a Multiskan Sky microplate spectrophotometer.

#### Fluorescence Quantification in Culture

The same culture, handling, and visualization conditions used for cytotoxicity evaluation of the PC12 cell line were applied in this assay. Cells were detached with 20% trypsin in PBS. After 24 h of growth, the medium was replaced with one of the following treatments: medium alone, medium with 0.02% DMSO–DMF, or medium containing 3QnCN at concentrations of 6.25, 12, 25, 50, or 100 μM. The cells were then incubated for another 24 h, after which treatments were removed and the cells washed with 50 μL PBS. An additional 50 μL of PBS was added before fluorescence was measured using an LS-55 spectrofluorometer (PerkinElmer).

#### Fluorescence Assay for Imaging

Slides were coated with poly-l-lysine (100 μg/mL) and incubated for 2 h, after which 200,000 cells were seeded and cultured for 24 h. Cells were then stimulated with Aβ_1–42_ (3.2 μM) for 12 h. Treatments were subsequently applied: (i) 3QnCN at 25 or 50 μM for 24 h; (ii) 3QnCN at the same concentrations for 1 h; (iii) 0.05% for 30 min. This served as the positive control, with 0.02% DMSO–DMF as a solvent for 3QnCN and water for ThS. Following treatment, the cells were washed with PBS and fixed in 4% formaldehyde for 30 min at 4 °C and then washed again with PBS. Nuclei were stained with DAPI for 10 min at room temperature in the dark, followed by six PBS washes. Slides were mounted and sealed. Imaging was performed on a confocal microscope (Nikon A1R HD25, Nikon ECLIPSE Ti2) using Alexa 488 antibody parameters to visualize the 3QnCN signal. Images were analyzed in FIJI, where the fluorescence intensity was quantified in four fields. Results were normalized to the blank, and statistical analysis was performed using one- way ANOVA, followed by Tukey test in GraphPad Prism 8.

## Conclusions

Utilizing quinoline as an electron-donating group within a π-conjugated architecture presents a promising strategy for developing fluorescent markers that target Aβ_1–42_, with the goal of labeling plaques associated with the early stages of Alzheimer’s disease. In this study, four quinoline-derived compounds were synthesized, and among them, 3QnCN demonstrated the most favorable photophysical properties, including a higher molar extinction coefficient, a smaller Stokes shift, and a higher quantum yield. Theoretical analyses indicated that 3QnCN possesses efficient ICT, with electron density effectively distributed along the molecule’s backbone. It reached one of the best ΔG values and was shown to be noncytotoxic at concentrations ranging from 3.1 to 100 μM. Its interaction with Aβ_1–42_ was quantitatively assessed through fluorescence assays, both in plate format and through direct observation. The 3QnCN signal was successfully visualized using fluorescence microscopy, revealing a green signal around the nucleus in cell cultures stimulated with Aβ_1–42_. Therefore, 3QnCN is proposed as a potential marker for Aβ_1–42_ in *in vivo* studies, which could serve as an asset in accelerating the development of new molecular design strategies for precise diagnosis and improved therapies for Alzheimer’s disease in its early stages.

## Supplementary Material



## Data Availability

All data are available in the manuscript and in the supporting material; however, if additional information is required, interested parties can obtain it by contacting the first author or the corresponding authors. Requests should be directed to the following email address: avictoria02sanmen@gmail.com or marcrh2002@yahoo.com.mx.

## Notes

Conditions of Aβ_1–42_ for direct interaction assay, which include an atomic force microscopy experiment; percentage of stained cells; chemical characterization, FT-IR (ATR), nuclear magnetic resonance (NMR), and mass spectrometry of 3QnCN, 2QnCN, 4QnBB, and 3QnB; (Figure S1) image analysis by AFM; (Figure S2) cell labeling with 3QnCN; (Figure S3) FT-IR spectra of 2QnCN, 3QnCN, 4QnBB, and 3QnB; (Figure S4) NMR of 3QnCN; (Figure S5) NMR of 2QnCN; (Figure S6) NMR of 4QnBB; (Figure S7) NMR of 3QnB; (Figure S8) ESI mass spectrum and fragmentation prediction of 3QnCN; (Figure S9) ESI mass spectrum and fragmentation prediction of 2QnCN; (Figure S10) ESI mass spectrum and fragmentation prediction of 4QnBB; (Figure S11) ESI mass spectrum and fragmentation prediction of 3QnB (PDF)
