# Supplementary material for: Design and Evaluation of Quinoline-derived Fluorophores for Labeling Amyloid Beta 1–42 in Alzheimer’s Disease
Source: ACS Omega. 2026 Jan 29;11(5):7343–58. doi: 10.1021/acsomega.5c07481 (PMC12903005; doi:10.1021/acsomega.5c07481)
Supplement: Supplementary file 1 [file ao5c07481_si_001.pdf]

Supporting information

Design and evaluation of quinoline-derived fluorophores for labeling amyloid beta 1-42 in Alzheimer's disease

Alma Victoria Sánchez-Mendoza[a,b], Rosa Angeles Vázquez-García[c*], Víctor Castaño[d], Mónica A. Torres-Ramos [e,f], Alan Hipólito Juárez-Solano[e,f], Raúl Horacio Camarillo-López[a], Martha Cecilia Rosales-Hernández[a]*

[a] Laboratorio de Biofísica y Biocatálisis, Sección de Estudios de Posgrado e Investigación, Escuela Superior de Medicina, Instituto Politécnico Nacional, Plan de San Luis y Díaz Mirón s/n, Ciudad de México 11340, México.

[b] División de Ingeniería en Nanotecnología, Universidad Politécnica del Valle de México, Av. Mexiquense, esq, Av. Universidad Politécnica s/n, Villa Esmeralda, Tultitlan, Estado de México 54910, México.

[c] Área Académica de Ciencias de la Tierra y Materiales, Universidad Autónoma del Estado de Hidalgo, Cd. Universitaria, Pachuca de Soto, Hidalgo 42184, México.

[d] Centro de Física Aplicada y Tecnología Avanzada, Universidad Nacional Autónoma de México, Boulevard 3001, Santiago de Querétaro, Querétaro 76230, México.

[e] Dirección de Investigación. Instituto Nacional de Neurología y Neurocirugía Manuel Velasco Suárez, Insurgentes sur 3877, La Fama, Tlalpan, Ciudad de México 14269, México.

[f] Centro de Investigación sobre el Envejecimiento. Centro de Investigación de Estudios Avanzados. Calz. de los Tenorios 235, Coapa, Granjas Coapa, Tlalpan, Ciudad de México 14330, México.

1. Conditions of Aβ_{1-42} for Direct Interaction Assay

1.1 Atomic Force Microscopy Assay

Dilutions of 5 μM of βA1-42 were prepared, and 5 μL of this solution was placed on a cm^2 coverslip and allowed to dry in a nitrogen atmosphere. Images were obtained using an atomic force microscope (MultiMode V connected to the NanoScope V controller, Bruker, USA), in tapping mode in air using RTESP probes (Bruker, USA). Two experiments were conducted to study the fibrils of the biological material, with images randomly obtained from different areas of each layer that composes the biological material. The images show fibrils accumulated throughout the sample (Fig. S3a), and image analysis reveals approximate heights of 10 μm (Fig. S3b). These results corroborate that after 48 hours of incubation, βA_{1-42} is found in fibrillar form.

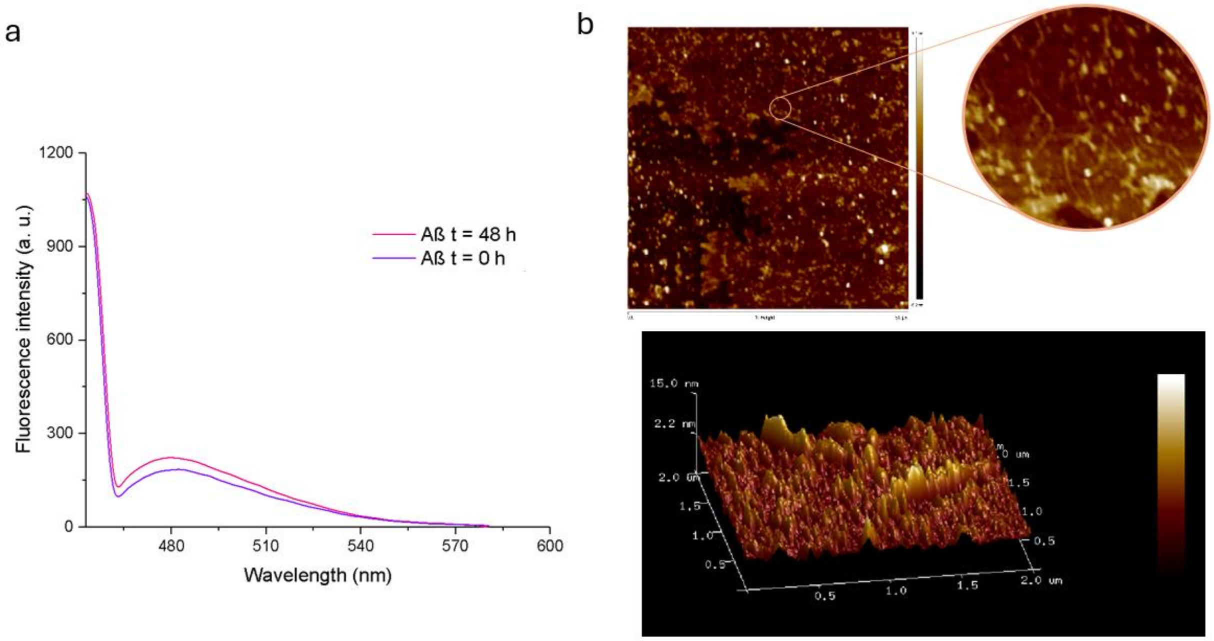

Figure S1. Image analysis by AFM: a) micrographs of βA_{1-42}, b) height analysis of βA_{1-42} fibrils.

2. Percentage of stained cell

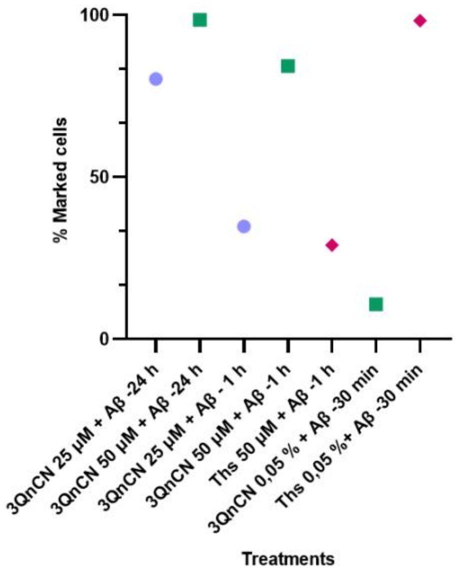

Figure S2. Cell Labeling with 3QnCN.

3. Chemical Characterization

3.1 FT-IR (ATR)

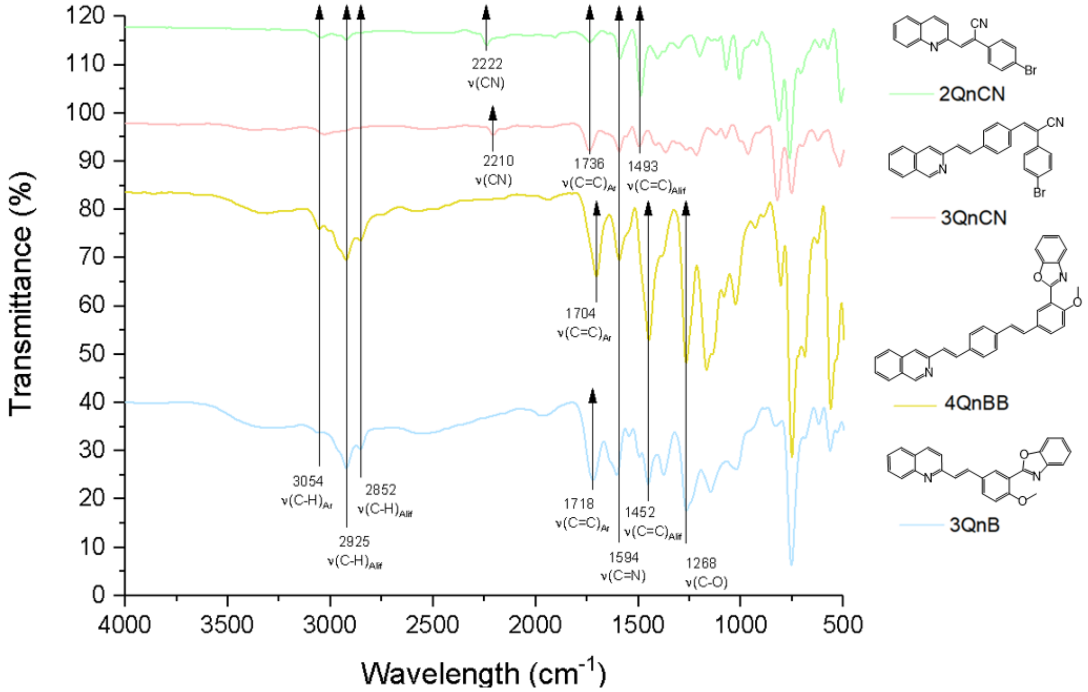

Figure S3. FT-IR spectra of 2QnCN, 3QnCN, 4QnBB, and 3QnB.

3.2 Nuclear Magnetic Resonance (NMR)

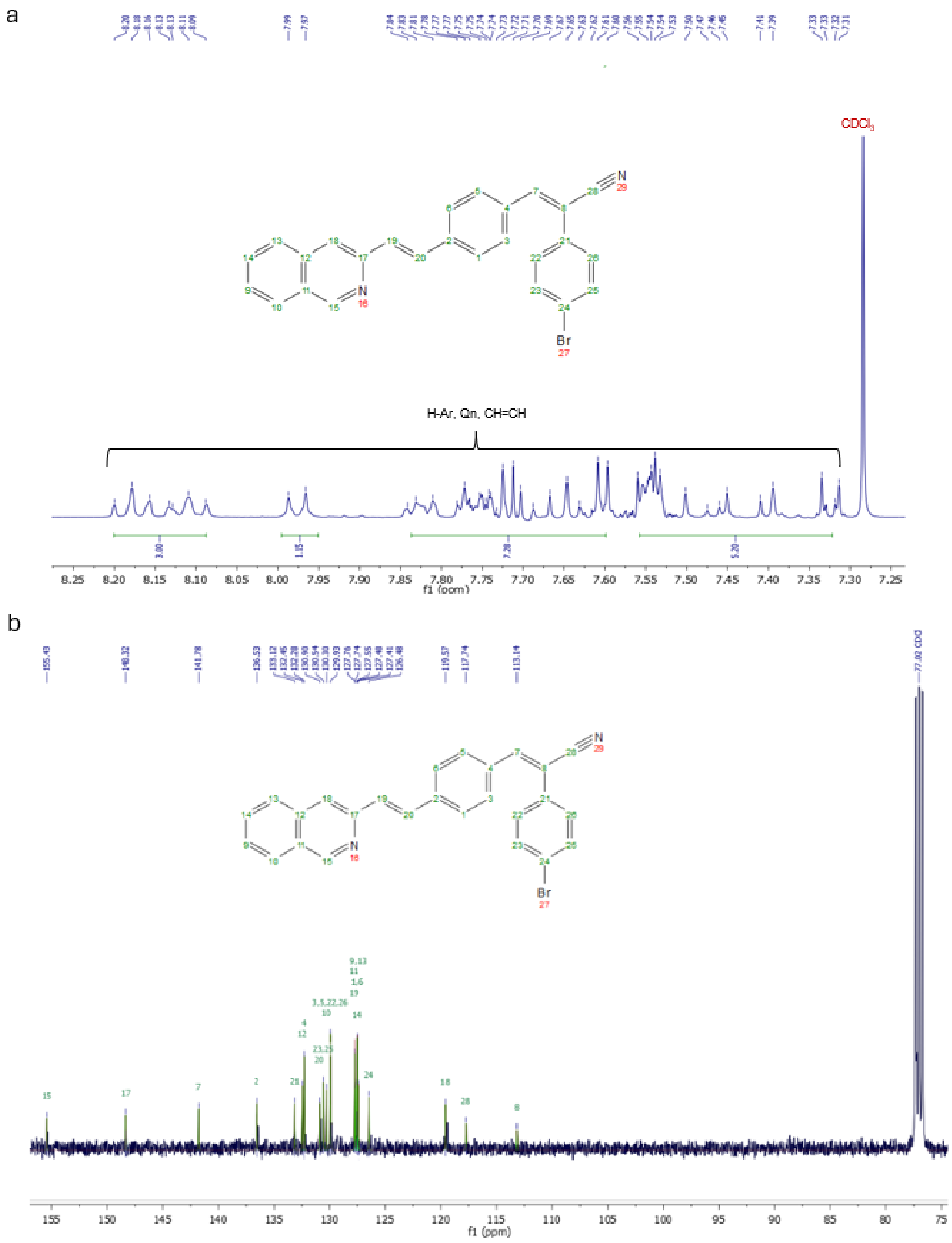

Figure S4. NMR of 3QnCN, a) ^{1}H; b) ^{13}C.

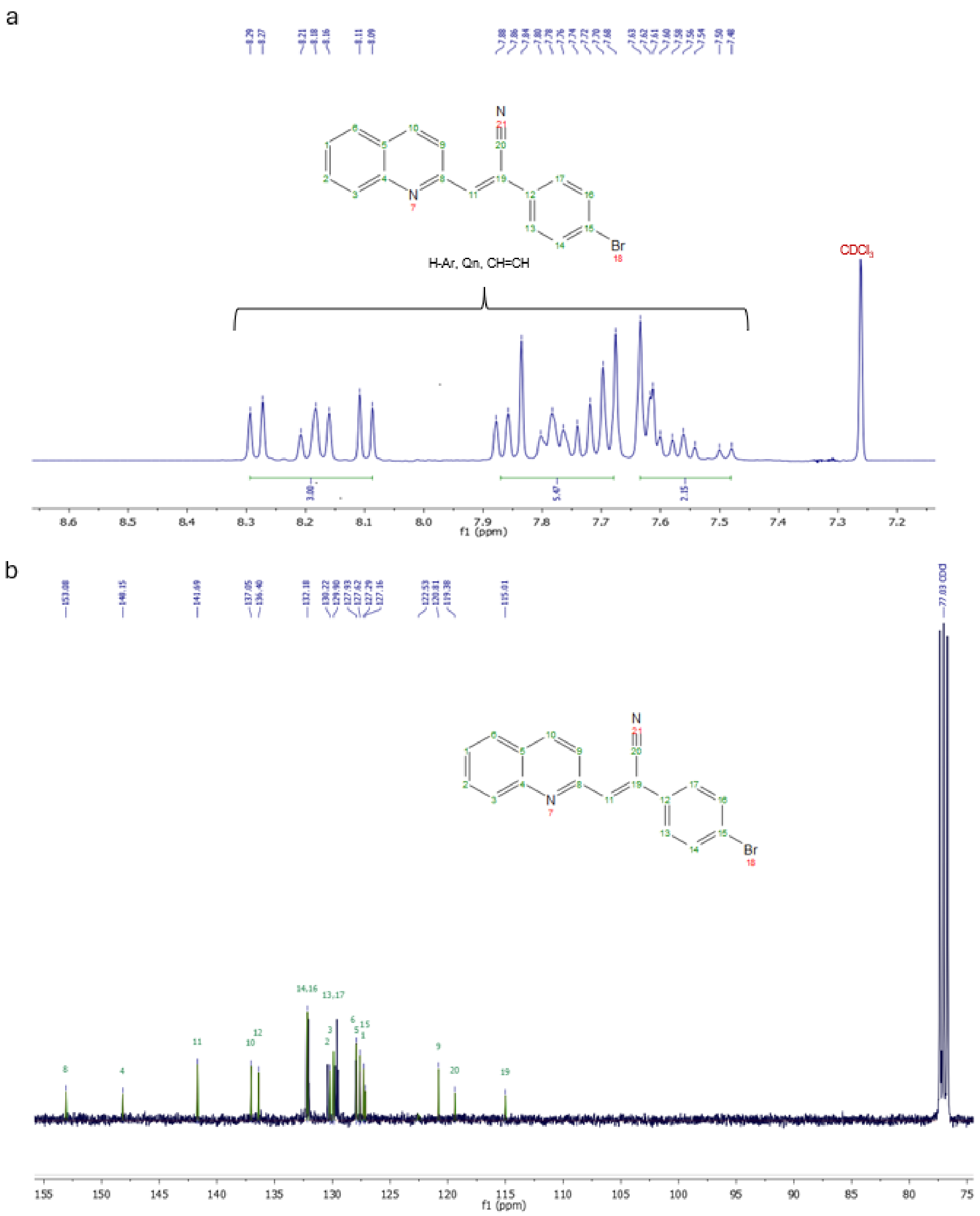

Figure S5. NMR of 2QnCN, a) ^{1}H; b) ^{13}C.

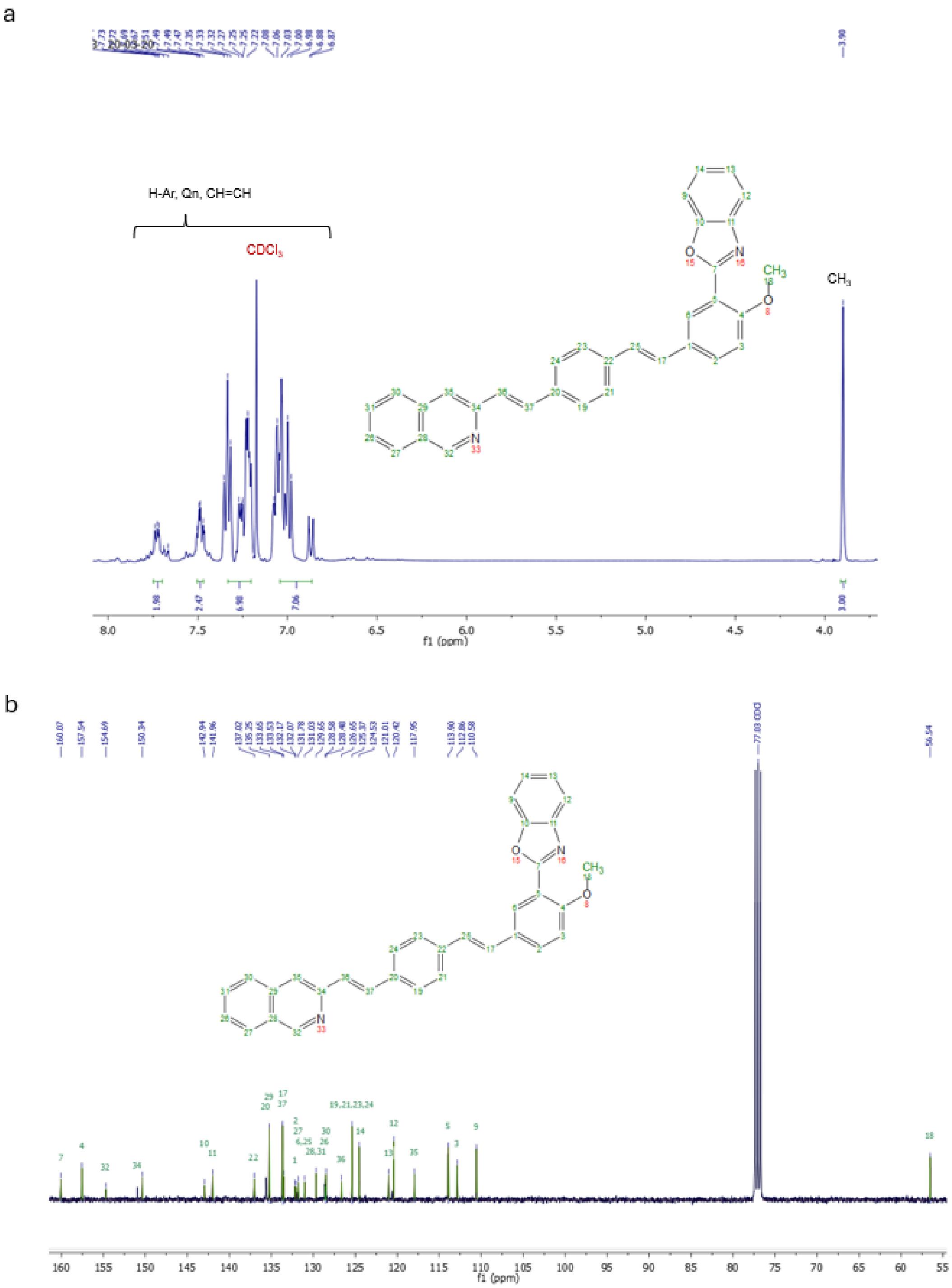

Figure S6. NMR of 4QnBB, a) ^{1}H; b) ^{13}C.

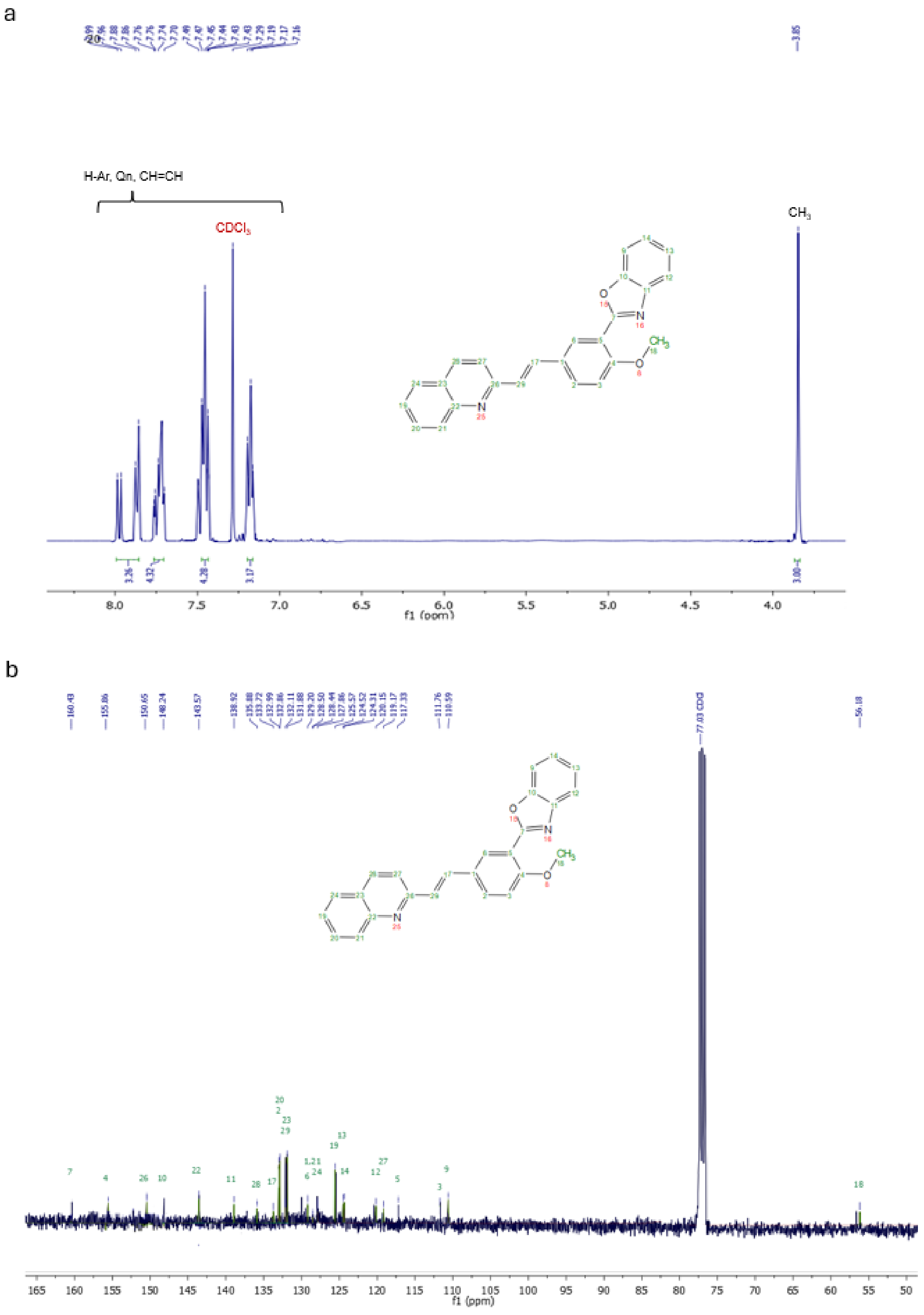

Figure S7. NMR of 3QnB, a) ^{1}H, ; b)^{13}C.

3.3 Mass spectroscopy of 3QnCN, 2QnCN, 4QnBB and 3QnB

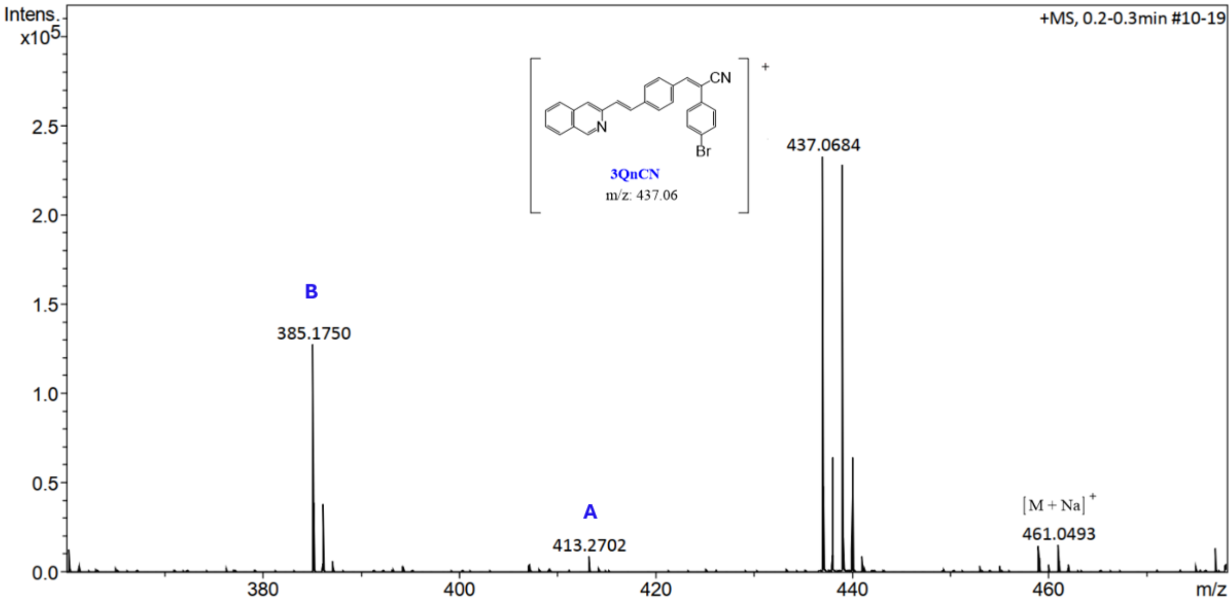

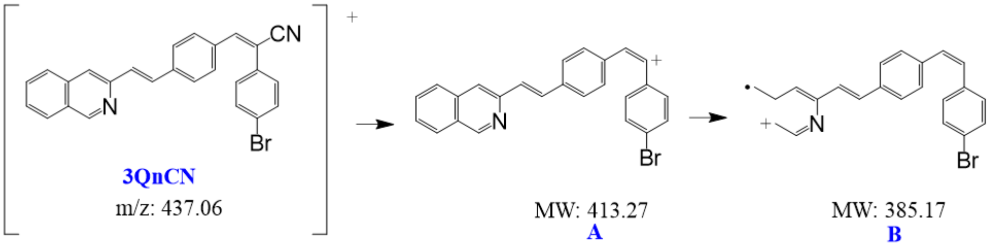

Figure S8. ESImass spectrum and fragmentation prediction of 3QnCN.

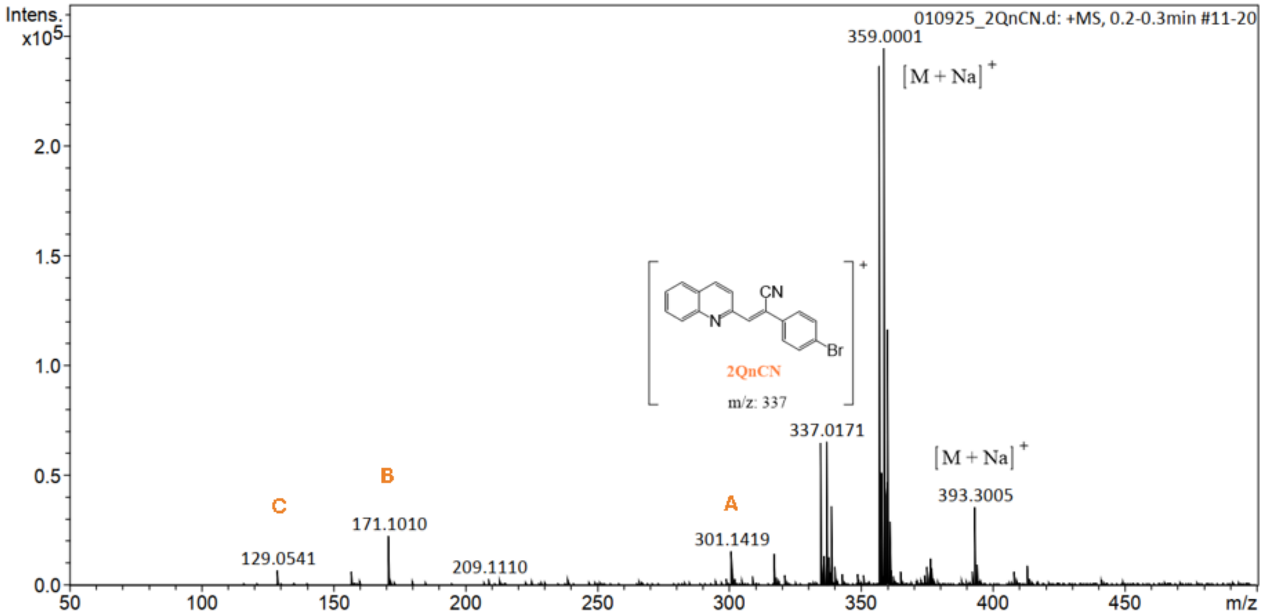

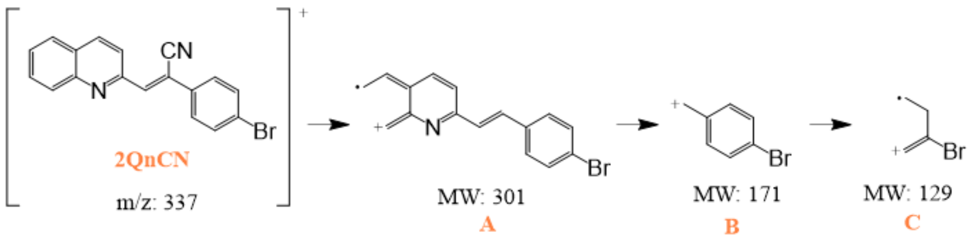

Figure S9. ESImass spectrum and fragmentation prediction of 2QnCN.

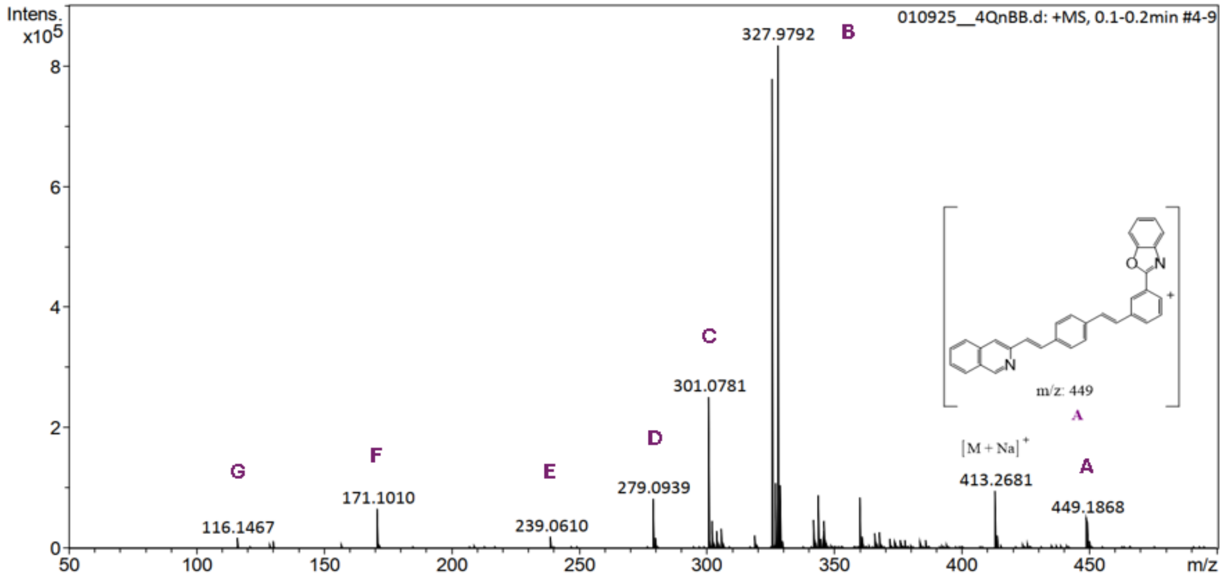

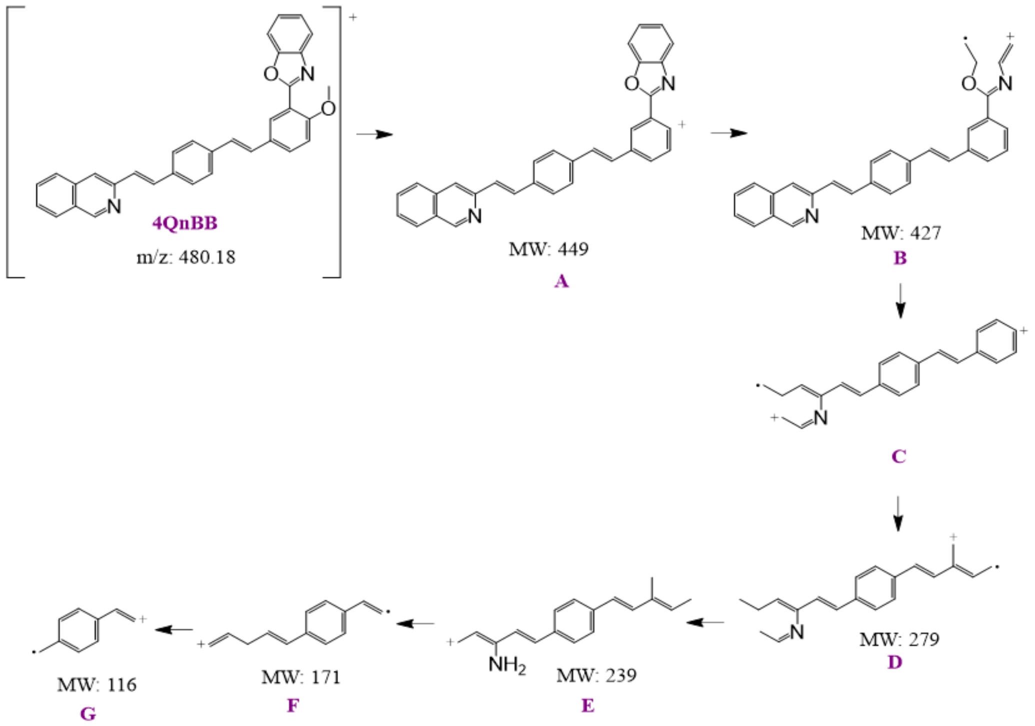

Figure S10. ESImass spectrum and fragmentation prediction of 4QnBB.

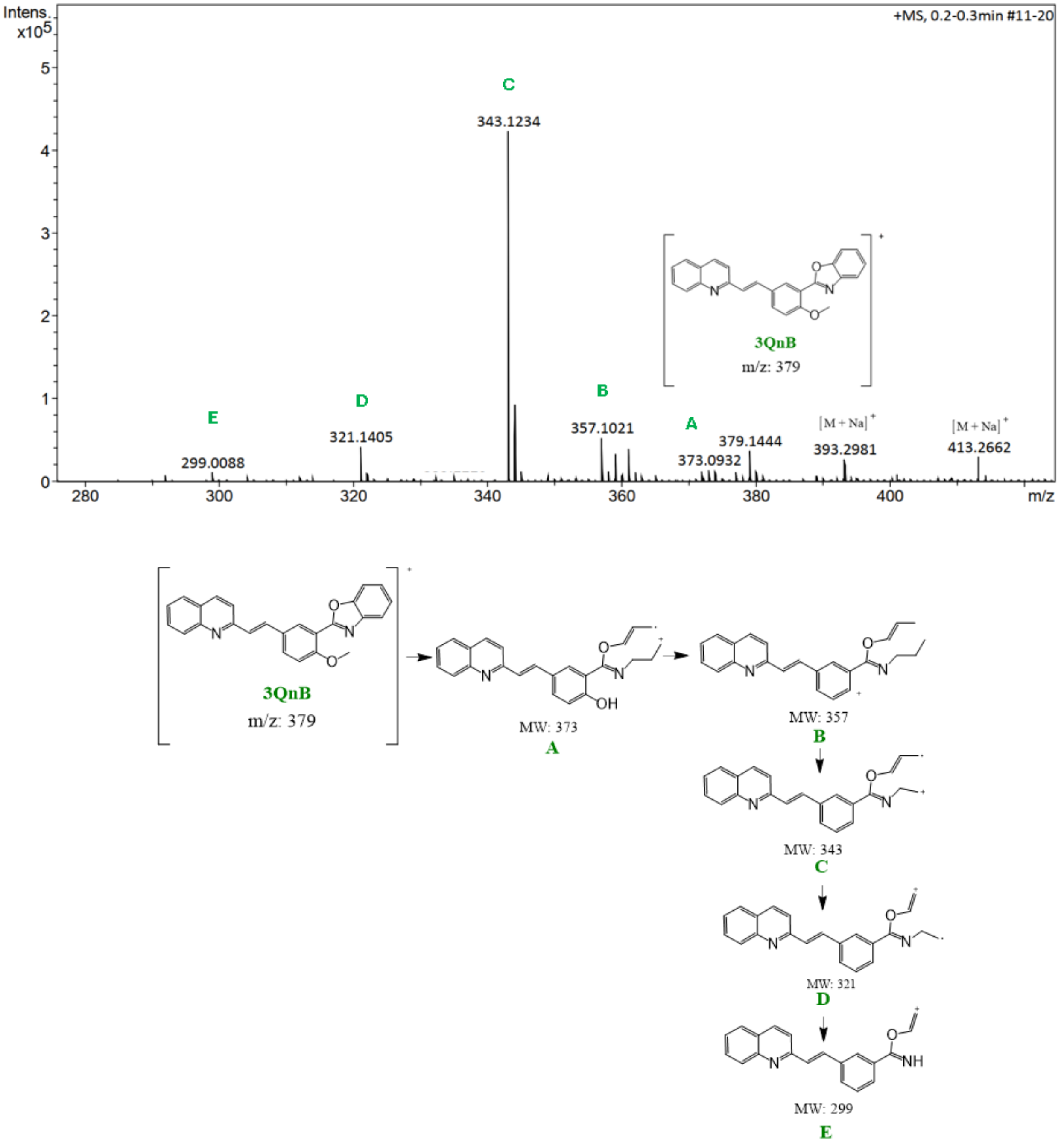

Figure S11. ESImass spectrum and fragmentation prediction of 3QnB.
